# Irreversibility of T-Cell Specification: Insights from Computational Modelling of a Minimal Network Architecture

**DOI:** 10.1371/journal.pone.0161260

**Published:** 2016-08-23

**Authors:** Erica Manesso, Hao Yuan Kueh, George Freedman, Ellen V. Rothenberg, Carsten Peterson

**Affiliations:** 1 Computational Biology and Biological Physics, Department of Astronomy and Theoretical Physics, Lund University, SE-223 62 Lund, Sweden; 2 Division of Biology, California Institute of Technology, Pasadena, CA 91125, United States of America; University of the Basque Country, SPAIN

## Abstract

**Background/Objectives:**

A cascade of gene activations under the control of Notch signalling is required during T-cell specification, when T-cell precursors gradually lose the potential to undertake other fates and become fully committed to the T-cell lineage. We elucidate how the gene/protein dynamics for a core transcriptional module governs this important process by computational means.

**Methods:**

We first assembled existing knowledge about transcription factors known to be important for T-cell specification to form a minimal core module consisting of TCF-1, GATA-3, BCL11B, and PU.1 aiming at dynamical modeling. Model architecture was based on published experimental measurements of the effects on each factor when each of the others is perturbed. While several studies provided gene expression measurements at different stages of T-cell development, pure time series are not available, thus precluding a straightforward study of the dynamical interactions among these genes. We therefore translate stage dependent data into time series. A feed-forward motif with multiple positive feed-backs can account for the observed delay between BCL11B versus TCF-1 and GATA-3 activation by Notch signalling. With a novel computational approach, all 32 possible interactions among Notch signalling, TCF-1, and GATA-3 are explored by translating combinatorial logic expressions into differential equations for BCL11B production rate.

**Results:**

Our analysis reveals that only 3 of 32 possible configurations, where GATA-3 works as a dimer, are able to explain not only the time delay, but very importantly, also give rise to irreversibility. The winning models explain the data within the 95% confidence region and are consistent with regard to decay rates.

**Conclusions:**

This first generation model for early T-cell specification has relatively few players. Yet it explains the gradual transition into a committed state with no return. Encoding logics in a rate equation setting allows determination of binding properties beyond what is possible in a Boolean network.

## Introduction

Gene regulatory networks (GRN) control multiple organismal and cellular processes including cell fate choices, metabolism, cell cycle, and signal transduction. The use of mathematical models to understand the architecture and function of GRN has become increasingly extensive, allowing us to rationalize results provided by experimental approaches and generate testable predictions [1].

Several influential studies concern the exploration of GRN characterising the immune system. For the B-cell lineage, experimental analysis was coupled with continuous models where gene expression intensities are described by ordinary differential equations [2, 3]. In contrast, the existing GRN models describing the T-cell lineage are mainly based on logical models, as most available data are only qualitatively robust [4–6]. Although network topologies were proposed, these models failed to account for the timing of watershed events in T-cell development, such as T-lineage commitment. Solving this problem is one of the goals of this work.

T cells are generated in the thymus, but their development requires a continuous source of new T-cell progenitors migrating from the bone marrow. T-cell progenitors entering the thymus have the ability to undertake not only T-cell fate, but also myeloid/dendritic, B, or NK cell fates [7, 8]. Through the first residence period in the thymus, these precursors are known as Early Thymic Precursors (ETP or DN1). Upon contact with Notch signalling [9, 10], these cells start to up-regulate genes distinctive for T-cell identity (DN2a stage), but still maintain the potential to become other cell types [11]. After initial lineage specification, cells undergo a subsequent transition into a lineage-committed state (DN2b), where major changes in their regulatory state take place [12]. Eventually they will rearrange their T-cell receptor genes primarily in the DN3a stage, and later [13], their post-commitment development depends on the outcome of these rearrangements. Successful commitment depends on BCL11B activation at the end of the DN2a stage and on the silencing of PU.1 shortly thereafter [14]. A kinetic explanation for the sharp changes in expression of these two genes can thus account for much of the timing of commitment. However, the known inputs involved in both gene expression changes appear to be present in the cells far earlier than commitment itself.

In this study, we analyse continuous GRN models to understand the control of T-cell lineage commitment timing in response to inductive signals. The circuit comprises only the genes encoding transcription factors that are established key-players in the specification process, *i.e*. TCF-1 (*Tcf7* gene), GATA-3 (*Gata3* gene) and BCL11B (*Bcl11b* gene) for the T-cell identity and PU.1 (*Spi1* gene) for the alternative fates [15]. We show that a feed-forward motif is sufficient to account for the delay between TCF-1 and GATA-3 activation by Notch signalling and the later activation of BCL11B. A novel computational approach is introduced here to explore different possible interactions among Notch signalling, TCF-1, and GATA-3, by translating 32 combinatorial logic expressions into differential equations for BCL11B production rate. The method consists of a preliminary exploration of the parameter space, where the derivatives of the time-series are estimated from changes in levels at different developmental stages, followed by parameter estimation by multi-objective optimisation [16]. To be able to identify the best combinatorial configurations, *in vivo* gene expression levels at different stages [17], data mapping Notch pathway activity [18, 19], and findings from *in vitro* gene perturbation experiments [11, 12, 20–22] are exploited. Our results reveal that only three combinatorial configurations explain the data, and these all predict a requirement for co-operativity in the GATA-3 regulation of BCL11B. This mechanism contemplates a coherent feed-forward motif that mediates input from Notch signalling to activate BCL11B through multiple layers of positive feedbacks involving TCF-1 and GATA-3. The resulting models can recapitulate (i) the deferral in T-cell commitment after exposure to Notch signalling, as controlled by activation of *Bcl11b*, and (ii) the irreversibility of the commitment process, which is established after BCL11B expression has saturated. Our approach for translating different combinatorial configurations into differential equations can easily be applied to other GRN.

## Materials and Methods

Our computational roadmap consisted of three steps: (i) Employ a minimal GRN, which has strong support in a series of experiments, for investigation; (ii) convert stage-dependent gene expression data to time-series for the genes involved in the GRN; (iii) map different logical interactions among these genes onto differential model equations and optimize the parameters by comparing model predictions with time-series data.

### GRN architecture

**Fig 1:**
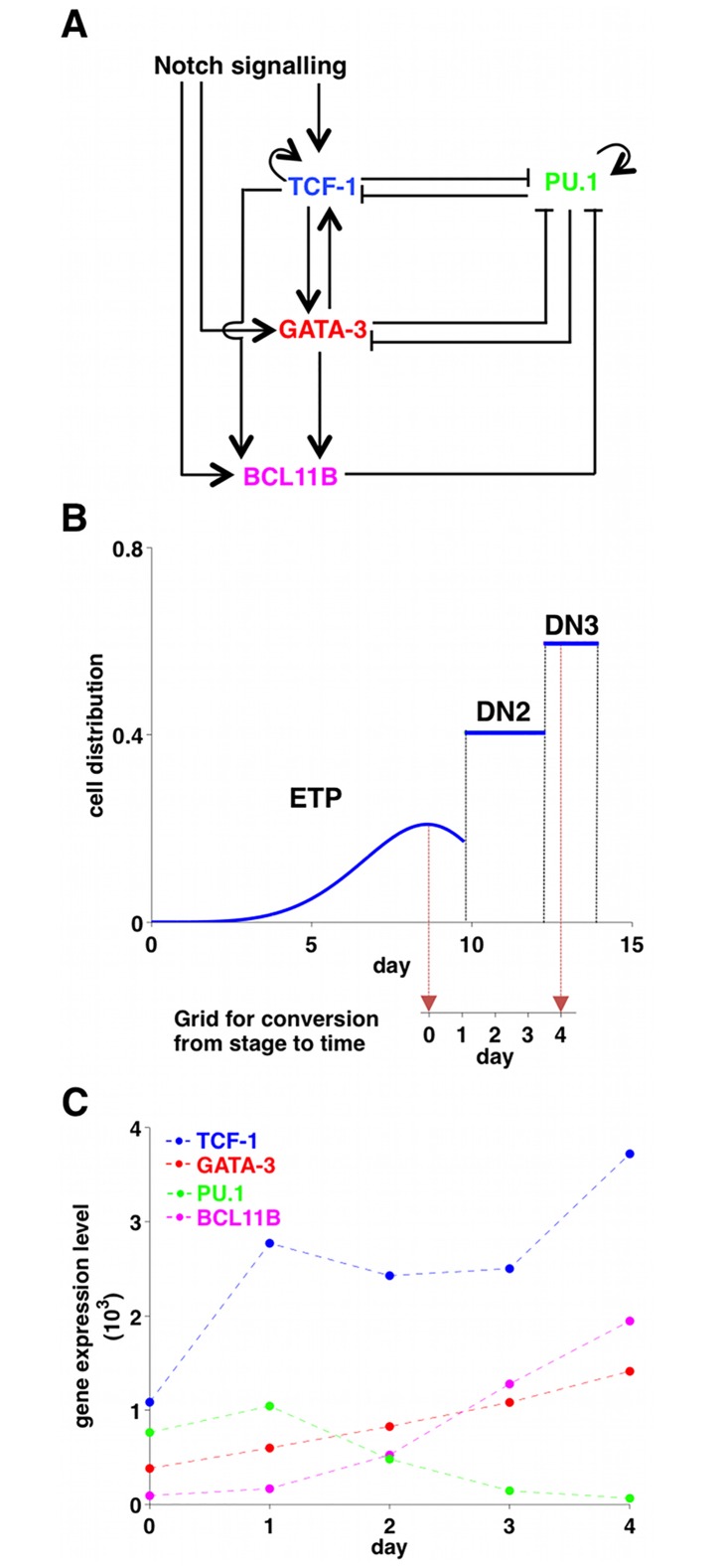
Core gene regulation in T-cell development. (A) A minimal gene regulatory network for T-cell specification. (B) From stage series to time series. In vivo cell distributions at ETP, DN2, and DN3 stages (adapted from [45]). The mean transit time in the ETP stage is 10 days circa, but most of the ETP cells are produced at the end of the 10 days, after several cell divisions: that justifies the lag of only 1 day between ETP (day 0) and ETP-DN2a (day 1). The mean transit time between DN2a and DN3a is about 3 days, so we decided to separate ETP-DN2a, DN2a (day 2), DN2b (day 3), and DN3a (day 4) by lags of 1 day each. (C) Gene expression profiles of TCF-1 (blue), GATA-3 (red), BCL11B (magenta), and PU.1 (green) at the fundamental stages of T-cell specification (adapted from [17] according to our conversion stage to time). The dashed lines were added to underline the trends of the gene expression profiles. The errors, which are shown in Fig 3 for each gene separately, were estimated (CV percentages) from for each population from the log values of relative expression.

The proposed GRN for T-cell specification (Fig 1A) is a reduced and updated version of a previously published circuit [6]. It includes the major features of early T-cell development: the induction of TCF-1, GATA-3, and BCL11B under the influence of Notch signal [23–27] and the presence of the feed-forward motif ending in BCL11B [25, 27–29]. While PU.1 and TCF-1 [20, 22, 30] and PU.1 and GATA-3 [11, 21, 22] are mutually capable of repressing each other’s expression, there is no evidence of the existence of a mutual inhibition between BCL11B and PU.1 [22], but it has been shown that BCL11B directly or indirectly terminates the expression of PU.1 [31]. On the other hand, there seems to be a mutual activation between TCF-1 and GATA-3 [27, 28]. BCL11B is then turned on both by TCF-1 [27] and by GATA-3 [28]. Self-positive interactions are known for PU.1 [32–34] and TCF-1 [27]. Notch signalling is fundamental for the activation of TCF-1 [25, 27], GATA-3 [22, 24, 35, 36], and BCL11B [20, 37], although its intensity is not necessarily uniform but rather it is known to increase from ETP to DN3a [19]. It is important to note that another factor, the complex Runx1-CBFbeta, has been implicated in both BCL11B activation and PU.1 repression [38–40]. However, because the levels of this complex change only slightly during T-cell commitment, and because two other Runx factors are expressed in a complementary developmental pattern [41], their contribution was assumed to be non rate-limiting.

### From stage-dependent data to time-series

Several studies have provided measurements of gene expression and RNA levels at different stages of T-cell development [11, 12, 17, 25, 41–44]; however, pure time-series are not available. We used the measurements for PU.1, TCF-1, GATA-3, and BCL11B expression at major stages of T-cell specification from Minguenau *et al*. [17]. These highly curated microarray data, which agree well with other studies that used qPCR, have the advantage of using a standard platform that covers most (if not all) of the entire genome. To translate these stage-dependent data into time-series, we used Mean Transit Times (MTT) that we calculated previously [45]. The strategy is shown in Fig 1B. The MTT in the ETP stage is ∼10 days. However, since the cells undergo multiple divisions before leaving the ETP population, most of the ETP cells at any given time are near the end of the 10 days [45]. This justifies approximating the interval as 1 day between the average ETP and ETP-DN2a cells. The MTT between DN2a and DN3a is about 3 days, so we decided to separate ETP-DN2a, DN2a, DN2b, and DN3a by intervals of 1 day each. The resulting time-series are shown in Fig 1C. Day 0 corresponds to the ETP stage, 1 to ETP-DN2a, 2 to DN2a, 3 to DN2b, and 4 to DN3a, respectively. The extracted time series were then subject to parametric smoothing.

### GRN models

The minimal GRN was translated into a set of ordinary differential equations where production rates were formulated according to the Shea-Ackers approach [46]. A constant degradation term was added to account for dilution due to cell division and protein degradation (S1 File). In the TCF-1 and GATA-3 differential equations TCF-1, GATA-3, and PU.1 were assumed to work as monomers. BCL11B was omitted since it is not expressed at all when GATA-3 and TCF-1 are first activated. The coherent feed-forward motif that mediates input from Notch signalling to BCL11B through TCF-1 and GATA-3, was tested in different configurations for the BCL11B production rate, related to all the possible combinatorial interactions among Notch signalling, TCF-1, and GATA-3 (32 in total, listed in Table 1). For example, an OR interaction was translated as follows:

$$\begin{array}{c} \frac{\kappa_{1}\text{Notch}+\kappa_{2}{\left[\text{TCF-1}\right]}^{n_{T}}+\kappa_{3}{\left[\text{GATA-3}\right]}^{n_{G}}}{1+\kappa_{1}\text{Notch}+\kappa_{2}{\left[\text{TCF-1}\right]}^{n_{T}}+\kappa_{3}{\left[\text{GATA-3}\right]}^{n_{G}}} \end{array}$$ (1)

where the brackets indicate the concentrations; *κ*_*i*_ are the kinetic parameters; *n*_*T*_ and *n*_*G*_ are the Hill coefficients, assuming the value of 1 in case of a monomer, 2 in case of a dimer. Note that no arbitrarily high Hill coefficients were introduced to force the system into switch-like behaviour in the absence of mechanism. We confined ourselves to BCL11B when it came to exploring the combinatorics as it has never been exploited before and it also hosts the endpoint of the important feed-forward motif. Needless to say, this also keeps the number of parameters low.

We have no quantitative information on how the Notch signalling behaves during the time interval investigated. However, it is estimated from Notch signal indicators (*Hes1*, *Dtx1*, *Il2ra*, *Ptcra* and *Nrarp*) that it increases between the ETP and DN3a stages by a factor up to 3.5 [19]. In general, Notch signalling activity can be represented as a right-skewed bell: it increases between ETP and DN3 stage and is then down-regulated [18, 19]. Since we were interested in the ETP to DN3a stages, we model the Notch signalling with a sigmoidal function.

All relevant rate equations involving expressions like Eq (1) are found in S1 File.

### Model selection—constraints and optimization

In general, the kinetic parameters contributing to the GRN dynamics were not measured previously and the time-series shown in Fig 1C have a relative paucity of data points. The equations for TCF-1, GATA3 and BCL11B contain in total 15 to 17 parameters and for PU.1 there are six. Hence, it was a challenge to achieve reliable and discriminative results for the different models by a straightforward fit to the data. We therefore imposed constraints from known features of the process, *e.g*. positivity of signals and concentrations and reasonable degradation rates to narrow down the parameter space.

**Fig 2:**
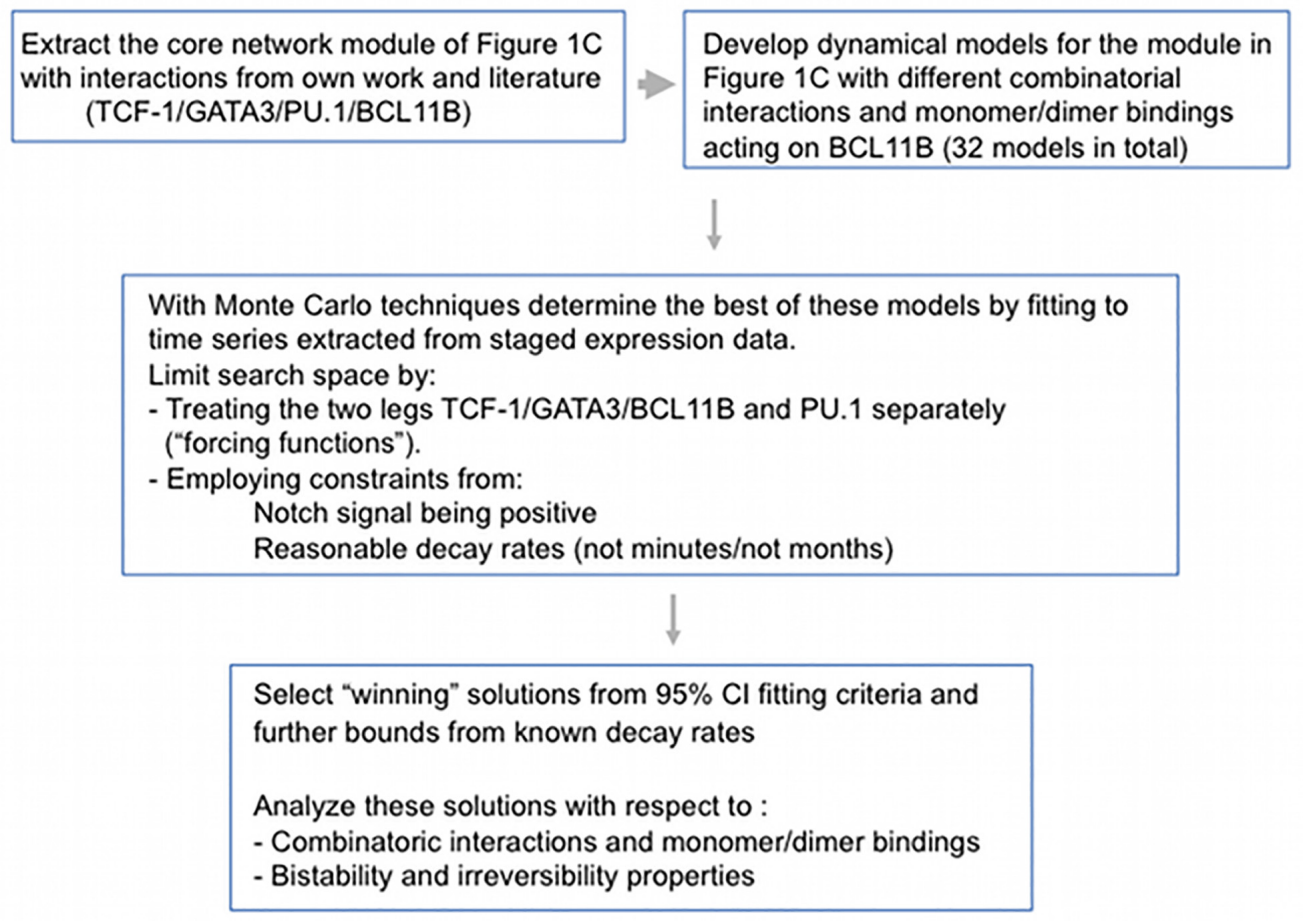
Workflow. Workflow from the selection of core module, extraction of gene expression of time series to fitting procedures and parameter estimation and selection for the TCF-1, GATA-3, and BCL11B dynamics.

After smoothing and calculation of the derivatives of the four expression profiles, we proceeded along two parallel paths to estimate the parameters: (A) fixing PU.1 from the smoothing procedure in the dynamic equations for TCF-1, GATA-3, and BCL11B and estimating the parameters for these equations; and (B) clamping TCF-1, GATA-3, and BCL11B to their smoothing profiles in the PU.1 dynamic equations and estimating the PU.1 parameter values. This “forcing function” procedure, which has been extensively used in successful physiological models [47], was employed to reduce the parameter space, *i.e*. enhancing the “a posteriori” identifiability. This approach is supported by the fact that PU.1 can be treated differently in the circuit because as long as the influence of Notch signalling is maintained, its activity has only modest repressive effect on TCF-1, GATA-3, or BCL11B levels [22, 48]. In detail we proceeded as follows (Fig 2 summarizes the overall workflow):

#### Constraints from Notch signalling

We rewrote the dynamical equations for TCF-1, GATA-3, and BCL11B separately to isolate the Notch signalling profile. The information related to the fold increase in Notch signalling implied a number of inequalities among the parameters describing the dynamics of TCF-1, GATA-3, and BCL11B, separately. These inequalities allowed us to define lower and upper parameter bounds (S1 File).

#### Optimisation of TCF-1, GATA-3, and BCL11B parameters

A multi-objective optimisation approach [16] with equal weights was applied to determine the best parameter configurations that fit simultaneously TCF-1, GATA-3, and BCL11B time-series (for each of the 32 combinatorial configurations of the BCL11B production rate), under the constraint that Notch signalling should increase between ETP and DN3a stage by a factor up to 3.5 [19].

#### Filtering the solutions

The resulting configurations were filtered according to two criteria: (1) the simultaneous fit of TCF-1, GATA-3, and BCL11B should be within the 95% intervals of confidence; and (2) plausible values for the half-lives of TCF-1, GATA-3, and BCL11B should be in the order of hours. In support to the later point, Cycloheximide (CHX) chase measurements of T-cell transcription factor half-lives in the pro-T cell like cell line, Adh.2C2 are reported in S1 Fig. While TCF-1 and BCL11B half-lives were measured in Del Real *et al*. [22], GATA-3 half-life was measured *de novo*. The 95% intervals of confidence were calculated considering a coefficient of variation in the data of 25% [17], thus accounting for data uncertainty.

#### Including PU.1

We expressed BCL11B profile as function of the kinetic parameters of PU.1 (S1 File). The requirement that BCL11B must be positive implied a number of inequalities that led to define most of the lower and upper bounds for the unknown parameters describing the dynamics of PU.1. A Monte Carlo method was applied to determine the missing bounds. Then, constrained simulated annealing (where we assumed that PU.1 has a lower inhibition power by BCL11B than by TCF-1 and GATA-3) followed by 95% intervals of confidence filtering provided *n* (order 10^2^) parameter values.

#### Simulating the entire network

To ensure reaching high levels of TCF-1, GATA-3, and BCL11B, but low levels of PU.1, the entire network was finally simulated to reach the steady state by taking separately each of the *n* parameter sets for the PU.1 dynamics described above. The parameters governing TCF-1, GATA-3, BCL11B, and Notch signals were fixed to their best values (*i.e*. minimising the mean squared differences between model prediction and data). Those parameter sets for PU.1 dynamics that guaranteed a reasonable steady state were selected, *i.e*. TCF-1, GATA-3 and BCL11B being high, while PU.1 is low.

### Bifurcation analysis

To investigate bistability and irreversibility, a bifurcation analysis was performed by varying the maximum value of Notch signalling (*N*, arbitrary unit) with the kinetic parameters set to the best ones in terms of adherence to the data.

### Measurement of protein half-lives

Half-lives of TCF-1, BCL1B, and GATA-3 proteins were measured by cycloheximide (CHX) chase experiments in the DN3-like cell line, Adh.2C2 [22]. Cells were treated with 10 *μ*g/mL of CHX for 0–8 hr of culture, then harvested at 2-hr intervals, and specific protein levels were determined in cell lysates by western blotting. Briefly, at the indicated times of culture, cells were collected by centrifugation, counted to determine viability, washed in PBS, and lysed by incubation on ice for 30 min, with occasional vortexing, in HEPES-buffered saline buffer (0.25 M NaCl, 50 mM Hepes pH 7.8) with 1% NP40 (IGEPAL), freshly supplemented with protease inhibitor cocktail. Then the lysates were cleared by centrifugation and the protein concentrations measured by modified Bradford assay (BioRad). Aliquots of 30 *μ*g of protein were loaded on each lane of 8% or 10% polyacrylamide gels for electrophoresis followed by western blotting. Primary antibodies used for western blots were: for GATA-3, BD PharmIngen 558686 (1/2500 dilution); for BCL11B, Bethyl Laboratories A300-383A (1/500 dilution); and for TCF-1, Cell Signaling Technologies, C63D9 (1/10,000 dilution), followed by Horseradish Peroxidase (HRP)-coupled goat anti-mouse or goat anti-rabbit secondary antibodies as appropriate. Chemiluminescent signals generated by HRP action on the detection reagent (Super Signal West Pico Plus, Thermo Fisher) were recorded by film and quantitated by densitometric scanning followed by ImageJ analysis.

Note that cycloheximide treatment resulted in significant toxicity, with no more than 50% of the cells still viable by 6 hr in most experiments, so that protein half-lives longer than this could have been underestimated. However, for GATA-3, a robust intracellular flow cytometric measurement assay we used previously to monitor levels of GATA-3 in different populations of primary cells [29] provided an additional, complementary method to determine protein levels per single cell where the measurements are electronically gated to consider only cells that are still viable. This method showed that GATA-3 levels dropped uniformly and unimodally in the population over a 6 hr CHX chase (S1A Fig). For this transcription factor at least, S1A and S1B Fig shows that the values calculated for GATA-3 on the basis of Mean Fluorescence Intensity values of viable cells (t_1/2_ ∼ 3 hr) are in good general agreement with the values calculated from the total population western blots (t_1/2_ ∼ 3–4 hr). S1C and S1D Fig shows that t_1/2_ values of the other two factors fall in a similar range and thus may also avoid strong effects of later viability loss.

## Results

### A limited number of network configurations accounts for the timing of BCL11B induction by a coherent feed-forward motif

Notch signaling, PU.1, GATA-3, and TCF-1 are essential regulatory drivers of the first stages of T-cell development, which culminate in lineage commitment when the BCL11B gene is finally activated. While experimental tests have shown the effects of perturbing each of the factors individually on the levels of expression of all other factors, the system dynamics that explain the overall developmental sequence have not been calculated before. Here, we established a model architecture based only on experimentally determined pairwise regulatory relationships [10–27] (Fig 1A) and tested its ability to work predictively to account for the time courses of changes in gene expression that mark the early stages T-cell development. Within this model framework, not only was each gene’s expression a function of the expression of multiple others, but also the precise logic between input pairs and valency of each kind of input at each target gene would affect the model’s behavior. Different variants of the model were therefore tested with different combinatorial regulatory rules for the control of BCL11B itself.

We compared 32 possible variants of the model containing different combinatorial expressions for controlling BCL11B production rates, accommodating the Notch signalling increase between ETP and DN3a stage by a factor up to 3.5 and testing for the ability to satisfy simultaneously the following conditions:

1. Providing a good simultaneous fit of TCF-1, GATA-3, and BCL11B time-series within the 95% intervals of confidence. Since almost all models contain the same number of parameters there was no added value in using the Akaike index for model discrimination.
2. Plausible values for the half-lives (order of hours) of TCF-1, GATA-3, and BCL11B.

**Fig 3:**
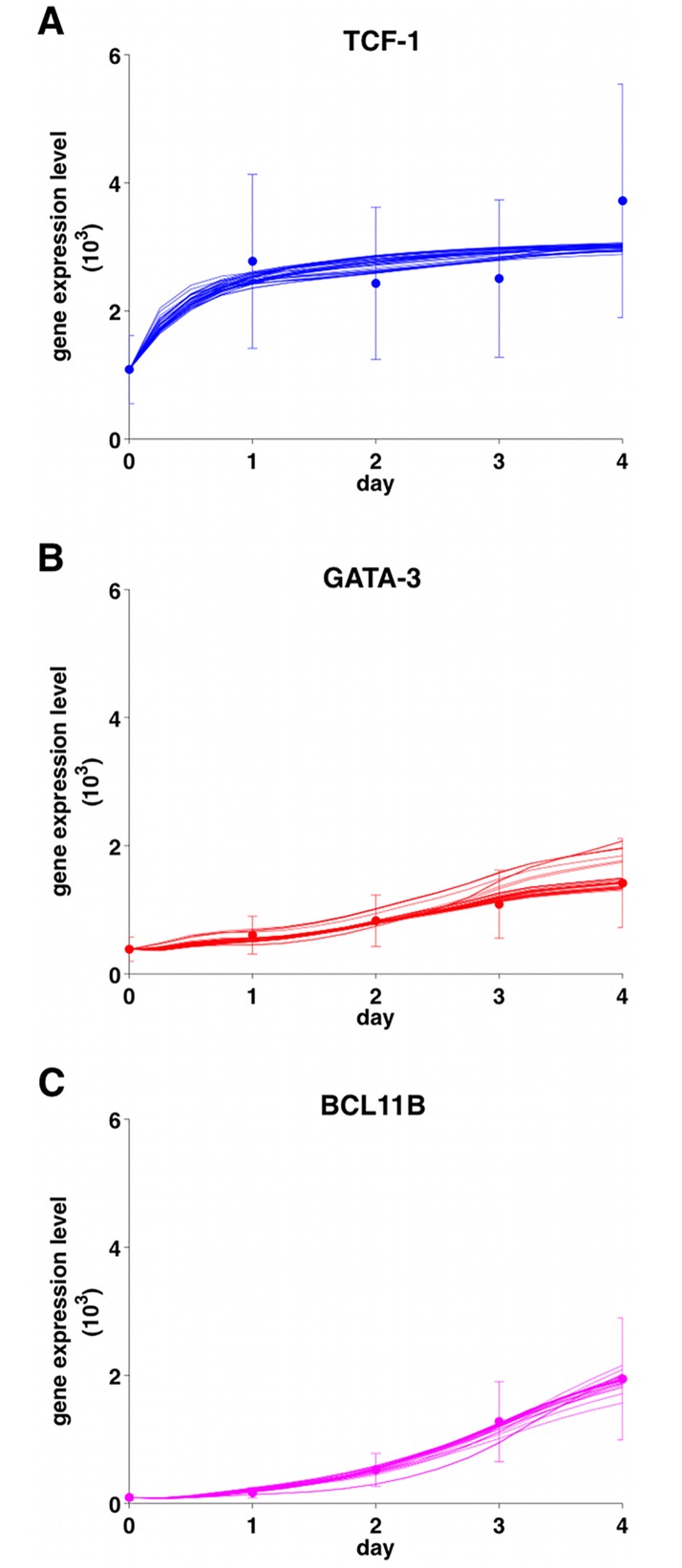
Model predictions. Realizations for the *n* parameter sets for the PU.1 dynamics (see text) of the winning model 6d, *i.e*. *dimer TCF-1* AND *(Notch* OR *dimer GATA-3)*: predictions for TCF-1 (panel A), GATA-3 (panel B), and BCL11B (panel C). Dots: data points (adapted from [17] according to our conversion stage to time); bars: 95% intervals of confidence. The errors were estimated (CV percentages) from for each population from the log values of relative expression [17].

Table 1 summarizes the results of the different selection steps for all the 32 configurations: only four combinations satisfied the above conditions. Fig 3 depicts how model predictions for one of the winning combinations are in line with the data (similar results were obtained for the other winning combinations, as shown in S2, S3 and S4 Figs). The estimated increases in Notch signalling between ETP and DN3a were only ∼1-1.4 (S5 Fig). For comparison we also report in S6 Fig the model predictions of a bad configuration.

It is clear that the minimal core architecture of Fig 1 and its model is able to account for deferral of turning on BCL11b from Fig 3C. This is due to the coherent feed-forward motif ending in BCL11B. Such motifs are known to cause time-delays, depending upon the parameter values [49].

The four winning combinations had in common GATA-3 acting on BCL11B as a dimer, and showed that at least one AND logic interaction is also needed between Notch signalling and TCF-1 or GATA-3 to give rise to the observed deferral of commitment.

The parameter values for the winning combinations are reported in Table 2. Notch signalling action was stronger on TCF-1 than on GATA-3 and on GATA-3 than on BCL11B (*i.e*. *η*_1_ > *δ*_1_ > *κ*_1_). The affinity constants of PU.1 for binding to the *Gata3* and *Tcf7* loci are calculated to be 10^2^-10^3^ fold stronger on GATA-3 (*δ*_3_) than on TCF-1 (*η*_4_). This prediction is in accordance with the ChiP-seq data from Zhang and colleagues [12] shown in S7 Fig. The mean and standard deviation of the final parameter values for PU.1 dynamics are summarized in S1 File. PU.1 inhibition was stronger on GATA-3 than on TCF-1, but GATA-3 had a stronger inhibitory effect than TCF-1 on PU.1. The mean half-life of PU.1 was estimated as roughly 4 hours. S8 Fig shows that the model predictions for PU.1 dynamics describe PU.1 expression data very well. The winning models are provided in SBML format (S2, S3 and S4 Files).

### The minimal model is sufficient to explain how T-cell specification becomes an irreversible process

**Fig 4:**
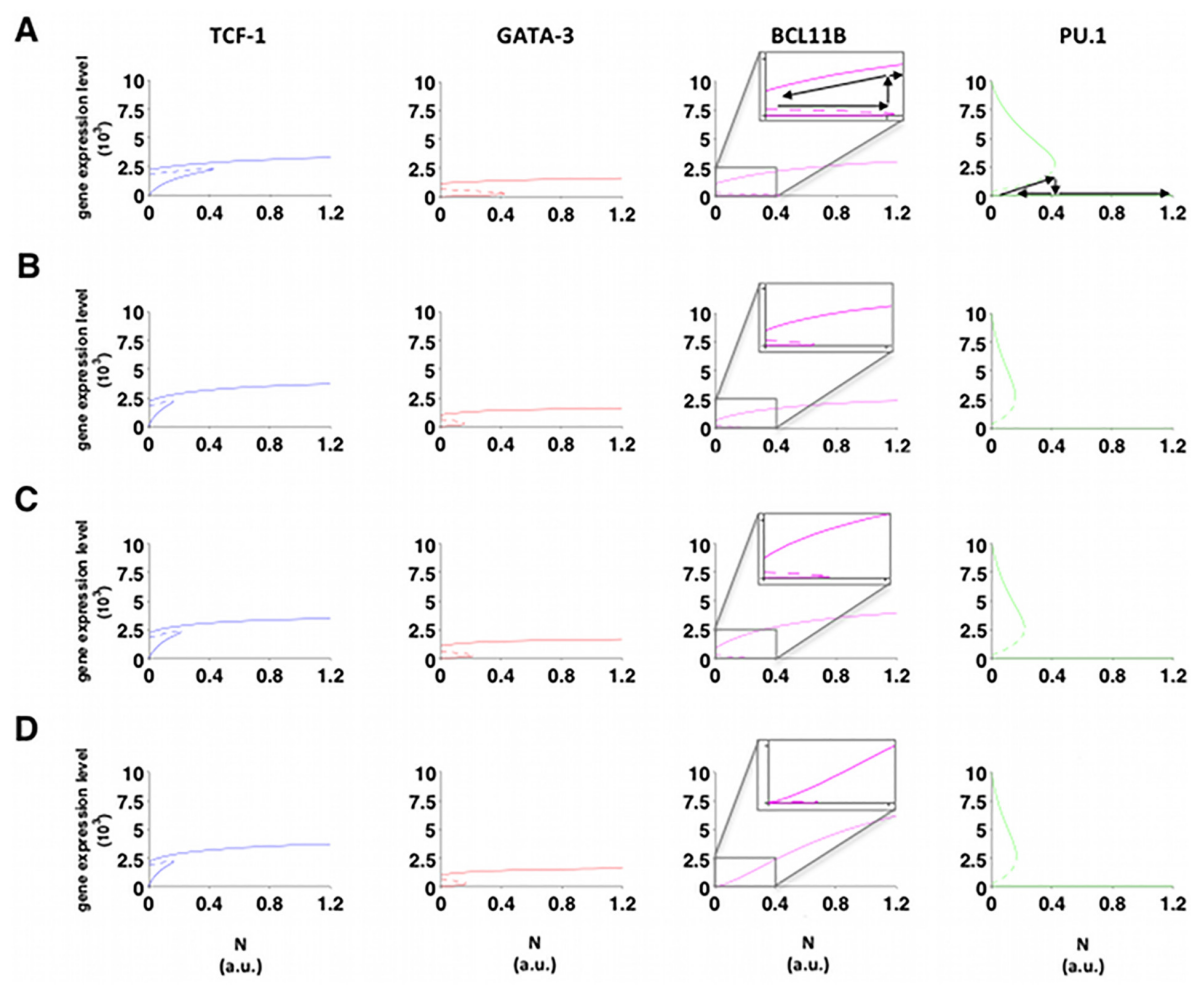
Bifurcation analysis. Bifurcation analysis with respect to the maximum value for the Notch signalling for the winning combinatorial configurations: (A) *dimer TCF-1* AND *(Notch* OR *dimer GATA-3)*; (B) *dimer GATA-3* AND *(Notch* OR *TCF-1)*; (C) *dimer GATA-3* AND *(Notch* OR *dimer TCF-1)*; (D) *Notch* AND *TCF-1* AND *dimer GATA-3*. Blue lines: TCF-1; red lines: GATA-3; magenta lines: BCL11B; green lines: PU.1. Continuous lines: stable states; dashed lines: unstable states. For BCL11B the bifurcation region is enlarged for the bistability regions in the different solutions. For both BCL11B and PU.1 arrows are drawn along the paths when increasing or decreasing the maximum Notch value.

T-cell specification becomes irreversible once the cells cross the commitment threshold [50], and its maintenance becomes Notch-independent after DN3a stage. To determine which winning configurations that also describe the critical point when this process becomes irreversible, a bifurcation analysis was performed by running the simulation backwards from PU.1 low and TCF-1, GATA-3, and BCL11B high starting conditions by reductions from the maximum level of Notch signalling (N). The resulting profiles for TCF-1, GATA-3, BCL11B, and PU.1 are shown in Fig 4. Decreasing N to zero only shuts off BCL11B in one combination where Notch signalling, TCF-1, and GATA-3 worked in a simple AND logic to activate BCL11B. In contrast, none of the models reactivate PU.1, and in the other three winning combinatorial configurations the models show full irreversibility.

**Table 1:** Model predictions for the different combinatorial configurations for BCL11B production after the selection based on the 95% confidence interval (CI) and plausible values for the half-lives. For each of the 8 interaction variants, Eqs 3-10 (S1 File), all combinations of the Hill coefficients of TCF-1 and GATA-3 (*i.e*. *n*_*T*,*B*_ and *n*_*G*,*B*_) were considered: (M,M); (M,D); (D,M); and (D,D) where M denotes monomer (Hill coefficient equals to 1) and D for dimer (Hill coefficient equals to 2). Notation: × indicates failure and ✓ success with regard to the CI 95% selection and ✓! those configurations that also give rise to reasonable values for the half-lives.

		Logics for BCL11B production	TCF-1	GATA-3	95% CI selection
1	a b c d	Notch OR TCF-1 OR GATA-3	M M D D	M D M D	× ✓ × ×
2	a b c d	(Notch AND TCF-1) OR GATA-3	M M D D	M D M D	× ✓ × ✓
3	a b c d	(Notch AND GATA-3) OR TCF-1	M M D D	M D M D	× × × ×
4	a b c d	Notch OR (TCF-1 AND GATA-3)	M M D D	M D M D	× × ✓ ✓
5	a b c d	Notch AND (TCF-1 OR GATA-3)	M M D D	M D M D	× × × ✓
6	a b c d	TCF-1 AND (Notch OR GATA-3)	M M D D	M D M D	× ✓ ✓ ✓!
7	a b c d	GATA-3 AND (Notch OR TCF-1)	M M D D	M D M D	× ✓! × ✓!
8	a b c d	Notch AND TCF-1 AND GATA-3	M M D D	M D M D	× ✓! ✓ ×

**Table 2:** Means and Standard Deviations (SD) for the winning parameters of the TCF-1, GATA-3, BCL11B, and Notch combinations with notation from Table 1: 6d = dimer TCF-1 AND (Notch OR dimer GATA-3); 7b = dimer GATA-3 AND (Notch OR TCF-1); 7d = dimer GATA-3 AND (Notch OR dimer TCF-1); 8b = Notch AND TCF-1 AND dimer GATA-3. The parameters are fully described in S1 File.

Model (see Table 1)	6d	7b	7d	8b
Parameters	Mean ± SD	Mean ± SD	Mean ± SD	Mean ± SD
*η*_1_·*N*	1.7E+00 ± 3.6E-01	2.3E+00 ± 7.5E-01	5.5E+00 ± 1.7E+00	2.0E+00 ± 1.4E+00
*η*_2_	2.2E-04 ± 3.4E-05	1.5E-04 ± 4.1E-05	2.3E-04 ± 5.92E-05	3.1E-04 ± 3.9E-05
*η*_3_	3.3E-04 ± 2.3E-04	6.4E-04 ± 1.2E-04	7.4E-04 ± 1.2E-04	6.2E-04 ± 1.3E-04
*η*_4_	4.7E-05 ± 3.1E-05	3.4E-05 ± 2.2E-05	3.3E-05 ± 1.7E-05	8.1E-06 ± 4.5E-06
*f*_*T*_	1.3E+04 ± 3.3E+03	1.2E+04 ± 3.7E+03	1.3E+04 ± 2.6E+03	1.4E+04 ± 2.1E+03
*γ*_*T*_	2.4E-04 ± 6.8E-06	2.1E-04 ± 2.0E-05	2.5E-04 ± 6.8E-06	2.2E-04 ± 8.6E-06
*δ*_1_·*N*	3.0E-04 ± 1.9E-03	5.9E-06 ± 1.6E-05	1.4E-03 ± 3.4E-03	1.9E+00 ± 4.1E-01
*δ*_2_	3.2E-05 ± 1.1E-05	5.7E-05 ± 5.1E-06	1.4E-05 ± 2.83E-06	7.7E-04 ± 6.4E-05
*δ*_3_	1.8E-03 ± 6.3E-04	1.1E-03 ± 7.2E-05	1.2E-03 ± 5.9E-05	8.3E-03 ± 1.1E-03
*f*_*G*_	1.2E+05 ± 3.6E+04	5.9E+04 ± 8.7E+03	1.8E+05 ± 1.7E+04	7.4E+03 ± 0.00E+00
*γ*_*G*_	5.2E-05 ± 2.1E-05	1.0E-04 ± 7.1E-06	3.2E-05 ± 3.7E-06	5.7E-04 ± 4.4E-06
*κ*_1_·*N*	1.0E-10 ± 3.9E-26	1.0E-10 ± 1.4E-26	1.0E-10 ± 3.9E-26	1.0E-10 ± 1.4E-26
*κ*_2_	3.6E-14 ± 7.3E-16	2.7E-11 ± 2.5E-13	1.1E-14 ± 5.47E-16	- ± -
*f*_*B*_	9.2E+03 ± 1.5E+03	1.2E+04 ± 8.0E+02	1.2E+04 ± 1.8E+03	5.3E+04 ± 5.5E+04
*γ*_*B*_	1.9E-04 ± 1.9E-05	8.7E-05 ± 1.2E-07	7.5E-05 ± 9.7E-06	1.7E-04 ± 5.1E-05
*α*	5.2E-01 ± 3.7E-01	1.6E-01 ± 6.2E-02	2.1E-01 ± 9.8E-02	3.9E-01 ± 2.1E-01
half-life TCF-1 (hour)	5.6E+00 ± 1.1E+00	7.2E+00 ± 2.0E+00	5.6E+00 ± 1.25E+00	5.41E+00 ± 6.05E-01
half-life GATA-3 (hour)	3.0E+00 ± 4.4E-01	2.9E+00 ± 5.9E-01	3.0E+00 ± 5.04E-01	3.95E+00 ± 3.05E-02
half-life BCL11B (hour)	9.9E+00 ± 1.4E+00	1.7E+01 ± 1.2E+00	2.0E+01 ± 3.9E+00	7.2E+00 ± 1.0E+01

## Discussion

Combinatorial control of T-cell lineage commitment has been suggested in topological gene network models before [5, 6, 50], but it has remained uncertain until now whether the known inputs are sufficient to explain the asynchronous gene expression dynamics of commitment using realistic parameters. Starting out from a minimal architecture for T-cell commitment, we determined the best combinatorial configurations for a family of dynamical models to describe expression levels of four key-genes: TCF-1, GATA-3, BCL11B, and PU.1. These models included all 32 possible combinatorial interactions among TCF-1, GATA-3 and Notch signalling to describe the coherent feed-forward motif mediating input from Notch signalling to BCL11B through TCF-1 and GATA-3. For each model, parameter identification was achieved by imposing known features of T-cell commitment and selecting those parameters that provided model predictions within the 95% confidence region of the data and reasonable decay rates. Four models out of 32 recapitulated the measured dynamics, among which three showed the irreversibility of T-cell commitment seen experimentally for BCL11B. This BCL11B irreversibility has a dynamical origin. Whereas irreversible PU.1 repression is not surprising in view of the main network architecture discussed (it has no regulated positive inputs, see Eq S11), when addition of a constant positive input was also tested, *e.g*. from the PU.1 activator RUNX1, a similar PU.1 pattern emerged (S9 Fig). To our knowledge, this is the first time that the dynamics of transition to irreversibility in mammalian developmental processes have been modelled based upon real data. The irreversibility is not entirely surprising given the architecture of intertwined loops and some of the non-winning models indeed also exhibit this feature. However, these do not fit the data well nor do they imply realistic decay rates.

The three successful models have in common the following properties:

1. Notch signalling shows OR logic interaction with either GATA-3 or TCF-1. This is a fundamental requirement for irreversible lockdown of the T-cell fate, which agrees with experimental data [20, 30].
2. GATA-3 regulates BCL11B in a cooperative manner, likely as a dimer. The existence and significance of such dimer forms are confirmed by a crystallographic study on the GATA-3 C-finger [51].
3. At least one AND logic interaction is needed between Notch signalling and TCF-1 or GATA-3. Without this feature, the feed-forward motif would not give rise to the observed deferral of commitment.
4. The estimated degradation rates for TCF-1 and BCL11B are supported by experiments conducted in the pro-T cell like cell line, Adh.2C2 (S1 Fig) [22]. Also, the estimates of relative binding strengths are consistent with ChiP-seq data in cases where these have been measured [12].

Importantly, all the results above were obtained without tweaking the architecture or other inputs or arbitrarily raising Hill coefficients. At first sight, model extraction from such sparse data might seem impossible. However, dividing the optimization problem into two legs motivated by the nature of the problem and imposing constraints from Notch signalling and reasonable decay rates, effectively reduces the dimensionality of the problem.

Our goal was to establish a quantitative, realistic foundation for testing whether the key genes activated in early T-cell precursors could be sufficient to explain the impact of Notch signalling on T-lineage commitment. In view of the distinctive kinetics [45] and finely graded stage-dependence of transcription factor effects in this process [50], it was important to construct a continuous-valued dynamical model to test the sufficiency of known regulators for mediating T-cell commitment (S1 File). Our approach showed the complementary role of logic and dynamic modelling techinques to study GRN: while the first allowed to reveal that Notch signalling acts with either GATA-3 or TCF-1 with an OR logic to ensure irreversible T-cell fate commitment, and one AND gate is required between Notch and TCF-1 or GATA-3 to enable commitment, the latter permitted to capture the dimeric binding of GATA-3 in the regulation of BCL11B and the estimation of plausible degradation rates and binding strengths.

Our approach reveals that the combination of a coherent feed-forward motif and multiple layers of positive feedback at intermediate nodes is sufficient to recapitulate important features of T-cell specification such as the cascade on gene expression activation. This architecture is referred as “positive regulatory embrace” of transcription factors downstream of a transient activating signal in development to make cell transitions to a new stable state [52]. This is the first time it has been demonstrated to provide a particularly good mechanism to generate not only irreversibility, but also time delays. Key to this advancement was an approach based upon combinatorial explorations of different interactions within a continuous rate equation framework, which is easily generalized to other GRN.

It should be pointed out though that the time delay could also be due to epigenetic effects such as slow, and potentially cell-cycle-dependent, transitions of chromatin states that are not accounted for in this work due to lack of experimental data.

## Supporting Information

S1 Fig Cycloheximide (CHX) chase measurements of T-cell transcription factor half-lives in the pro-T cell like cell line, Adh.2C2 [22]. Cells were treated with 10 *μ*g/mL of CHX for 0–8 hr and analyzed by intracellular staining and flow cytometry (S1A Fig) or by western blotting from cell lysates (S1B–S1D Fig), as described in the Materials and Methods. Panel A: intracellular staining for GATA-3, carried out as in [29], gated on viable cells only. A1: histograms of intracellular GATA-3 staining intensity on a log10 scale as compared with background staining with control IgG (red curve). A2: plot of specific GATA-3 staining levels (Mean Fluorescence Intensities, IgG control background values subtracted) as a function of time of CHX treatment. Panel B: western blot of GATA-3 protein stability in an independent CHX chase experiment. As a negative control (non-T), whole cell lysate from the RAW264.7 macrophage cell line is shown. Panel C: analysis of CHX chase samples from western blots of BCL11B in three separate, independent experiments. Panel D: analysis of CHX chase samples from western blots of TCF-1 in three separate, independent experiments. Although insufficient to distinguish precisely between the stabilities of these three transcription factors, these results indicate that in this pro-T cell like cell line, t1/2 values for all three are in the range of 2–5 hr.
(PDF)

S2 Fig Winning model 7b, i.e. *dimer GATA-3* AND *(Notch* OR *TCF-1)*: predictions for TCF-1 (panel A), GATA-3 (panel B), and BCL11B (panel C). Dots: data points (adapted from [17] according to our conversion stage to time); continuous lines: model predictions; bars: 95% intervals of confidence.
(PDF)

S3 Fig Winning model 7d, i.e. *dimer GATA-3* AND *(Notch* OR *dimer TCF-1)*: predictions for TCF-1 (panel A), GATA-3 (panel B), and BCL11B (panel C). Dots: data points (adapted from [17] according to our conversion stage to time); continuous lines: model predictions; bars: 95% intervals of confidence.
(PDF)

S4 Fig Winning model 8b, i.e. *Notch* AND *TCF-1* AND *dimer GATA-3*: predictions for TCF-1 (panel A), GATA-3 (panel B), and BCL11B (panel C). Dots: data points (adapted from [17] according to our conversion stage to time); continuous lines: model predictions; bars: 95% intervals of confidence.
(PDF)

S5 Fig Notch signalling corresponding to the winning combinatorial configurations: (A) 6d, *dimer TCF-1* AND *(Notch* OR *dimer GATA-3)*; (B) 7b, *dimer GATA-3* AND *(Notch* OR *TCF-1)*; (C) 7d, *dimer GATA-3* AND *(Notch* OR *dimer TCF-1)*; (D) 8b, *Notch* AND *TCF-1* AND *dimer GATA-3*. (PDF)

S6 Fig Model predictions for a bad combinatorial configuration 1a, i.e. *Notch* OR *TCF-1* OR *GATA-3*: the predictions for TCF-1 and BCL11B are out of the intervals of confidence. Dots: data points (adapted from Mingueneau *et al*. [17] according to our conversion stage to time); continuous lines: model predictions; bars: 95% intervals of confidence. The predicted decay rate for BCL11B was around 24 days.
(PDF)

S7 Fig PU.1 in vivo shows higher occupancy of sites around the *Gata3* locus (A) than of sites around the *Tcf7* locus (encoding TCF-1) (B). Shown are UCSC browser tracks representing in vivo binding of endogenous PU.1 to these loci in developing T-cell precursors, based on ChIP-seq. Data presented are from the published study of Zhang *et al*. [12]. Cell populations analyzed in panels A and B are pro-T cells derived from fetal liver hematopoietic precursors by in vitro differentiation (FLDN1, FLDN2a, and FLDN2b) and also TCRa-deficient CD4+ CD8+ thymocytes (Thy DP) as a representative of later stages of T-cell development. RefSeq gene models from NCBI build 37 (mm9) are shown at the top of each panel. The red track near the top of each panel is the chromatin accessibility mark, H3K4me2, which is found at open enhancers and promoters, to locate active cis-regulatory elements. The bottom track in each panel represents mammalian sequence conservation as another landmark for potential regulatory element. The brown tracks between these reference tracks represent the PU.1 ChIP-seq peaks (reads/million) around the *Gata3* locus (A) and the *Tcf7* locus (B). The data in A and B are from the same ChIP tracks with identical y axis scales between them; PU.1 peak heights in the two panels are directly comparable. Note that PU.1 binding is generally similar in magnitude in DN1 and DN2a stages but declines in DN2b stage and disappears by DP stage. However, the number and occupancy of PU.1 sites is much greater around *Gata3* than around a similar region of *Tcf7*.
(PDF)

S8 Fig Winning model predictions for PU.1. Dots: data points (adapted from Mingueneau *et al*. [17] according to our conversion stage to time); continuous lines: model predictions; bars: 95% intervals of confidence.
(PDF)

S9 Fig Bifurcation analysis with respect to the maximum value for the Notch signalling for the winning combinatorial configuration 6d, i.e. *dimer TCF-1* AND *(Notch* OR *dimer GATA-3)*, when an additional constant positive input on PU.1 was tested. Blue lines: TCF-1; red lines: GATA-3; magenta lines: BCL11B; green lines: PU.1. Continuous lines: stable states; dashed lines: unstable states.
(PDF)

S1 File Supporting Text. (PDF)

S2 File SBML model for the configuration 6d. (XML)

S3 File SBML model for the configuration 7b. (XML)

S4 File SBML model for the configuration 7d. (XML)
