# Supplementary figures and images for: Irreversibility of T-Cell Specification: Insights from Computational Modelling of a Minimal Network Architecture
Source: PLoS One. 2016 Aug 23;11(8):e0161260. doi: 10.1371/journal.pone.0161260 (PMC4995000; doi:10.1371/journal.pone.0161260)

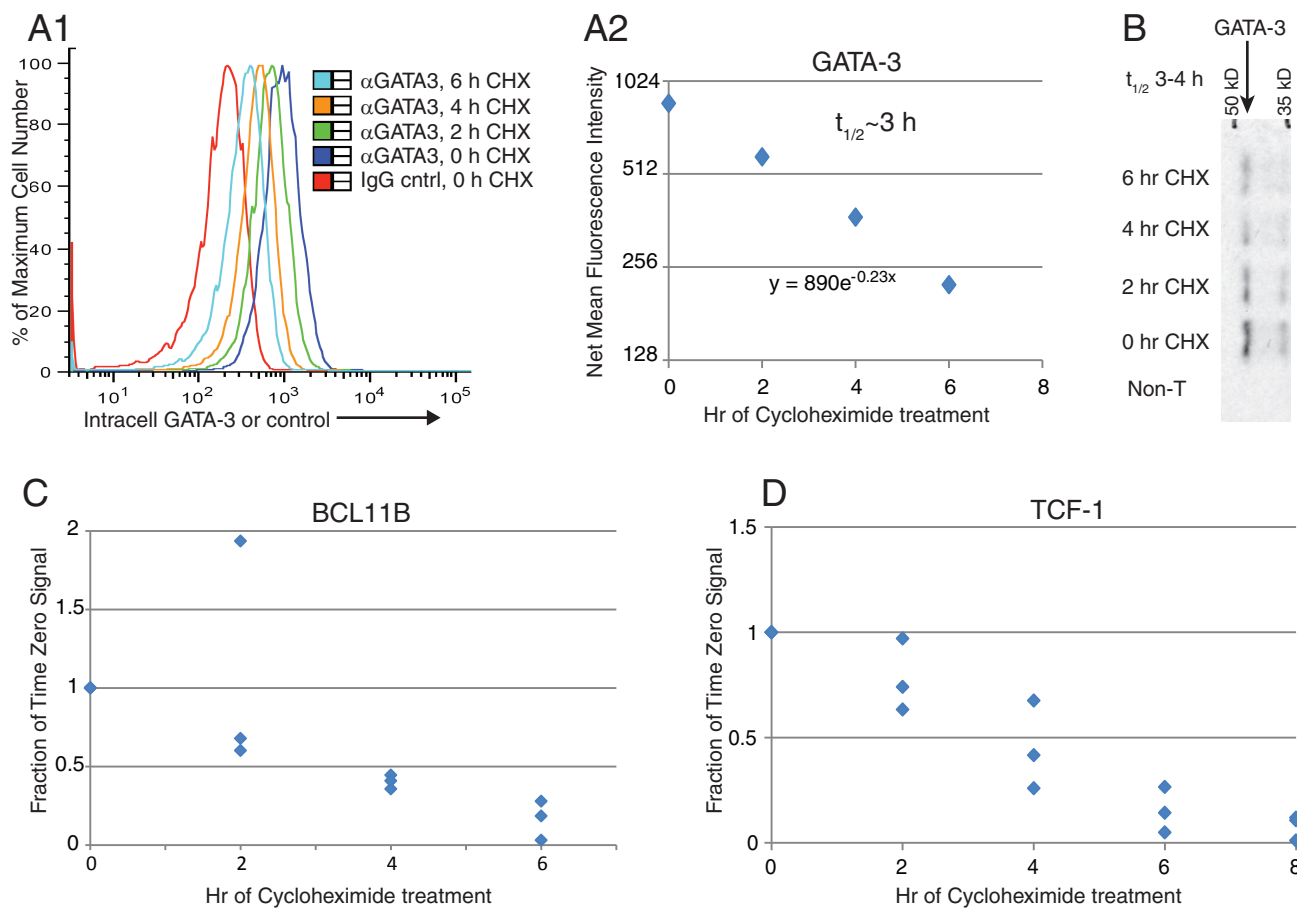

Figure S1

Supplement: S1 Fig — Cells were treated with 10 μg/mL of CHX for 0–8 hr and analyzed by intracellular staining and flow cytometry (S1A Fig) or by western blotting from cell lysates (S1B–S1D Fig), as described in the Materials and Methods. Panel A: intracellular staining for GATA-3, carried out as in [29], gated on viable cells only. A1: histograms of intracellular GATA-3 staining intensity on a log10 scale as compared with background staining with control IgG (red curve). A2: plot of specific GATA-3 staining levels (Mean Fluorescence Intensities, IgG control background values subtracted) as a function of time of CHX treatment. Panel B: western blot of GATA-3 protein stability in an independent CHX chase experiment. As a negative control (non-T), whole cell lysate from the RAW264.7 macrophage cell line is shown. Panel C: analysis of CHX chase samples from western blots of BCL11B in three separate, independent experiments. Panel D: analysis of CHX chase samples from western blots of TCF-1 in three separate, independent experiments. Although insufficient to distinguish precisely between the stabilities of these three transcription factors, these results indicate that in this pro-T cell like cell line, t1/2 values for all three are in the range of 2–5 hr. (PDF) [file pone.0161260.s001.pdf]

dimer GATA-3 AND (Notch OR TCF-1)

**A**

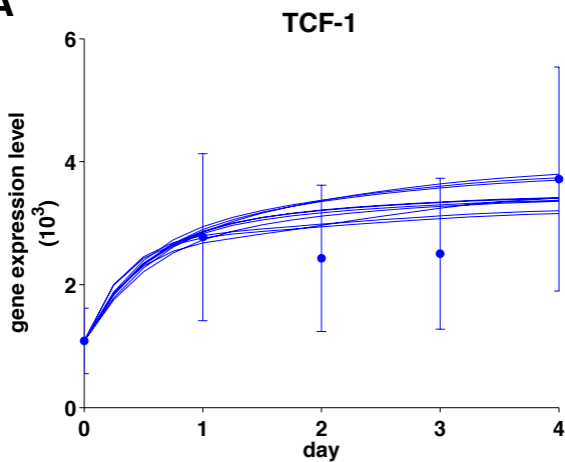

**B**

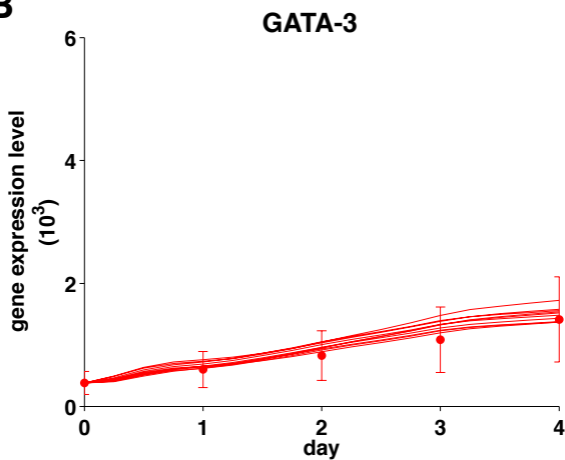

**C**

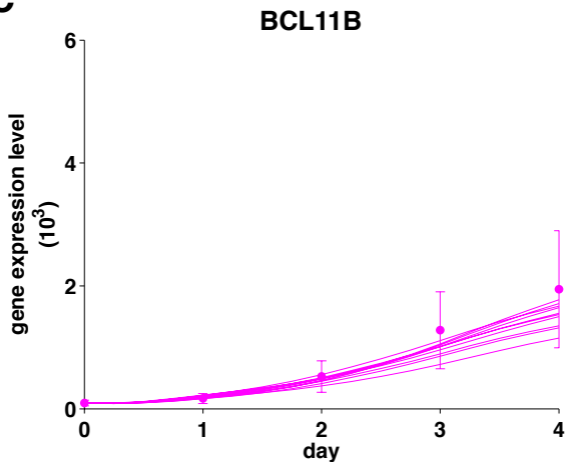

Supplement: S2 Fig — Dots: data points (adapted from [17] according to our conversion stage to time); continuous lines: model predictions; bars: 95% intervals of confidence. (PDF) [file pone.0161260.s002.pdf]

dimer GATA-3 AND (Notch OR dimer TCF-1)

**A**

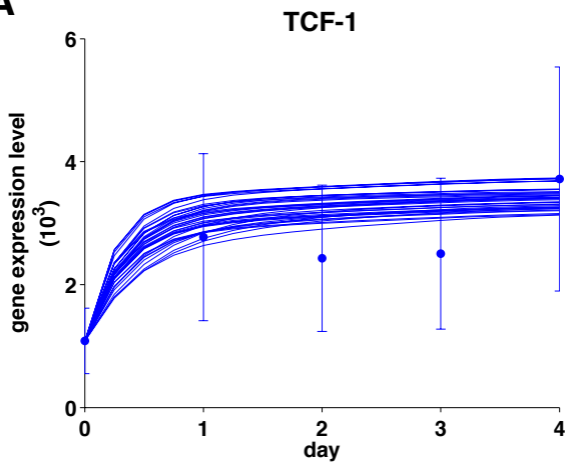

**B**

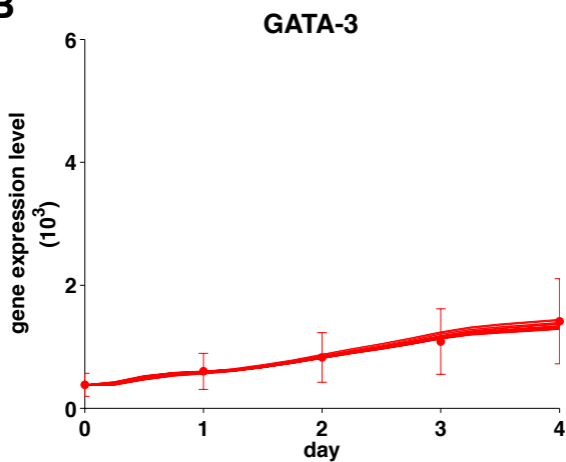

**C**

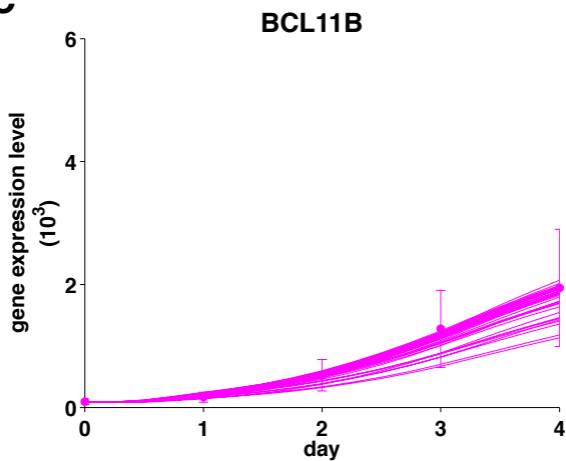

Supplement: S3 Fig — Dots: data points (adapted from [17] according to our conversion stage to time); continuous lines: model predictions; bars: 95% intervals of confidence. (PDF) [file pone.0161260.s003.pdf]

# Notch AND TCF-1 AND dimer GATA-3

**A**

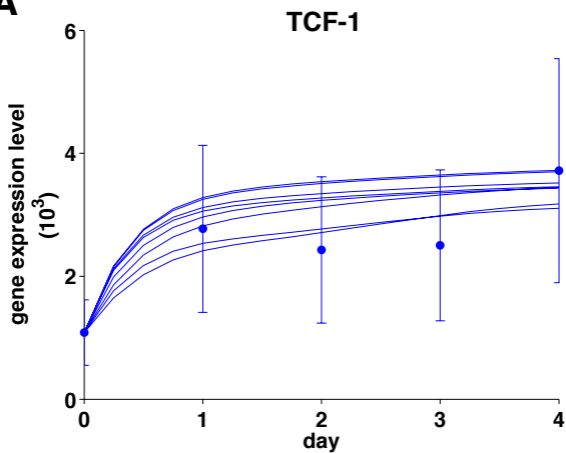

**B**

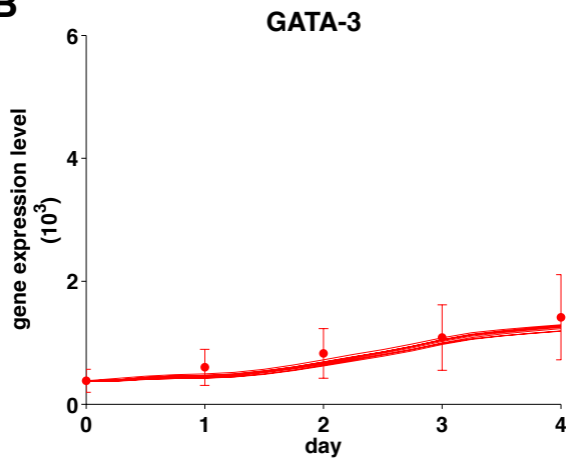

**C**

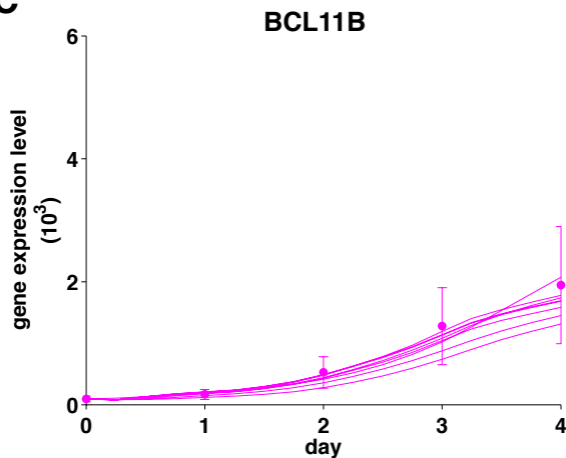

Supplement: S4 Fig — Dots: data points (adapted from [17] according to our conversion stage to time); continuous lines: model predictions; bars: 95% intervals of confidence. (PDF) [file pone.0161260.s004.pdf]

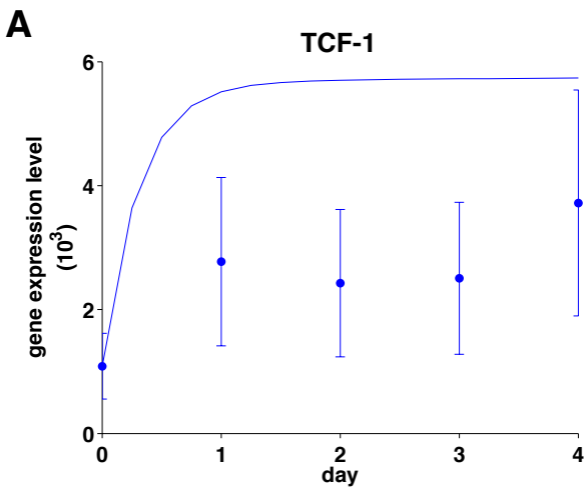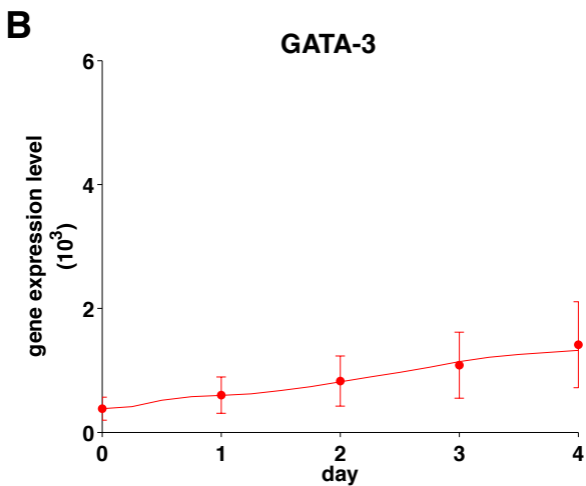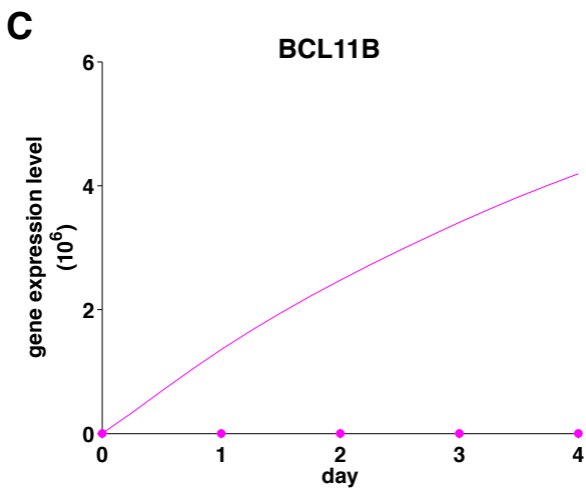

Supplement: S6 Fig — Dots: data points (adapted from Mingueneau et al. [17] according to our conversion stage to time); continuous lines: model predictions; bars: 95% intervals of confidence. The predicted decay rate for BCL11B was around 24 days. (PDF) [file pone.0161260.s006.pdf]

A

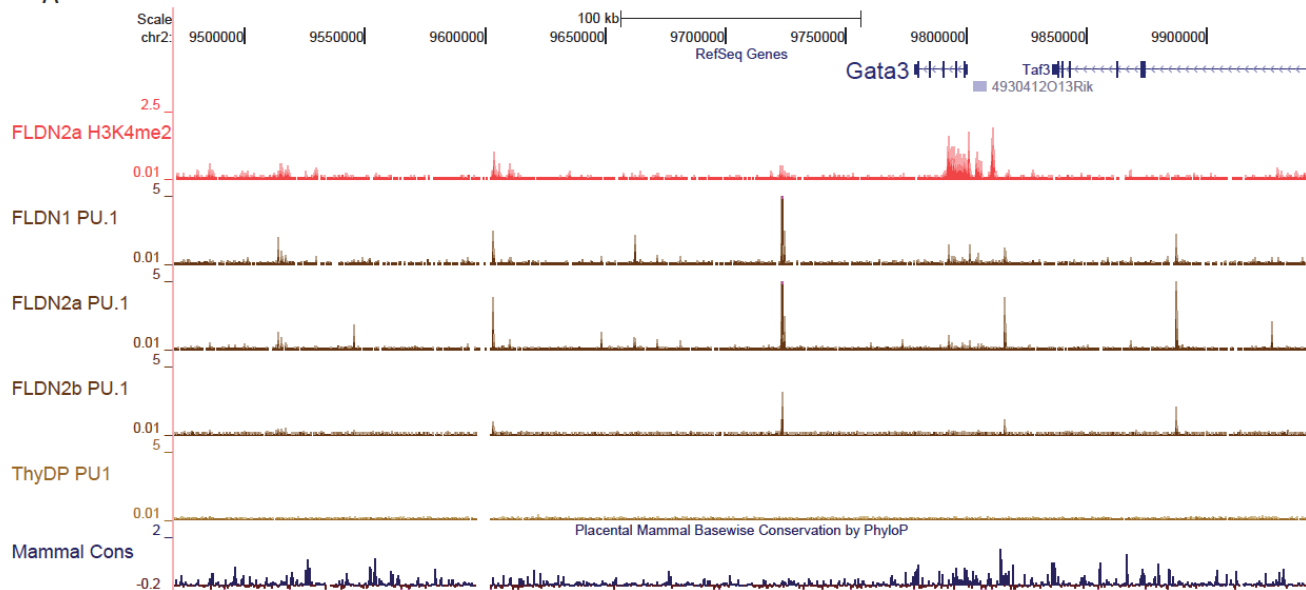

B

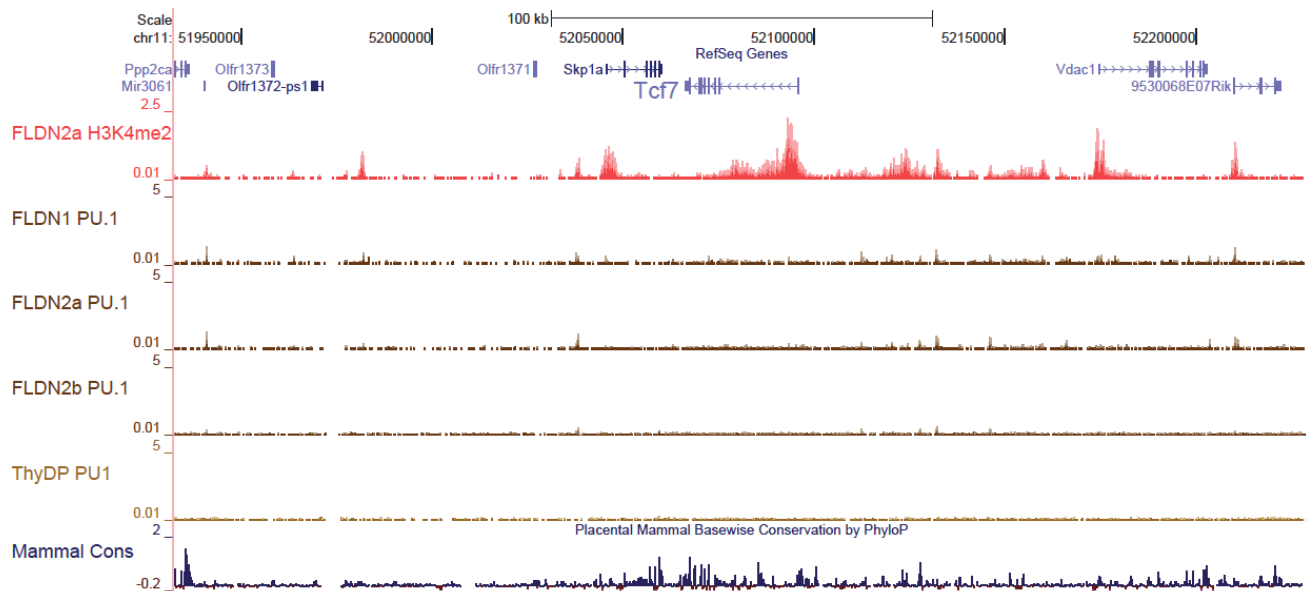

Supplement: S7 Fig — Shown are UCSC browser tracks representing in vivo binding of endogenous PU.1 to these loci in developing T-cell precursors, based on ChIP-seq. Data presented are from the published study of Zhang et al. [12]. Cell populations analyzed in panels A and B are pro-T cells derived from fetal liver hematopoietic precursors by in vitro differentiation (FLDN1, FLDN2a, and FLDN2b) and also TCRa-deficient CD4+ CD8+ thymocytes (Thy DP) as a representative of later stages of T-cell development. RefSeq gene models from NCBI build 37 (mm9) are shown at the top of each panel. The red track near the top of each panel is the chromatin accessibility mark, H3K4me2, which is found at open enhancers and promoters, to locate active cis-regulatory elements. The bottom track in each panel represents mammalian sequence conservation as another landmark for potential regulatory element. The brown tracks between these reference tracks represent the PU.1 ChIP-seq peaks (reads/million) around the Gata3 locus (A) and the Tcf7 locus (B). The data in A and B are from the same ChIP tracks with identical y axis scales between them; PU.1 peak heights in the two panels are directly comparable. Note that PU.1 binding is generally similar in magnitude in DN1 and DN2a stages but declines in DN2b stage and disappears by DP stage. However, the number and occupancy of PU.1 sites is much greater around Gata3 than around a similar region of Tcf7. (PDF) [file pone.0161260.s007.pdf]

**PU.1**

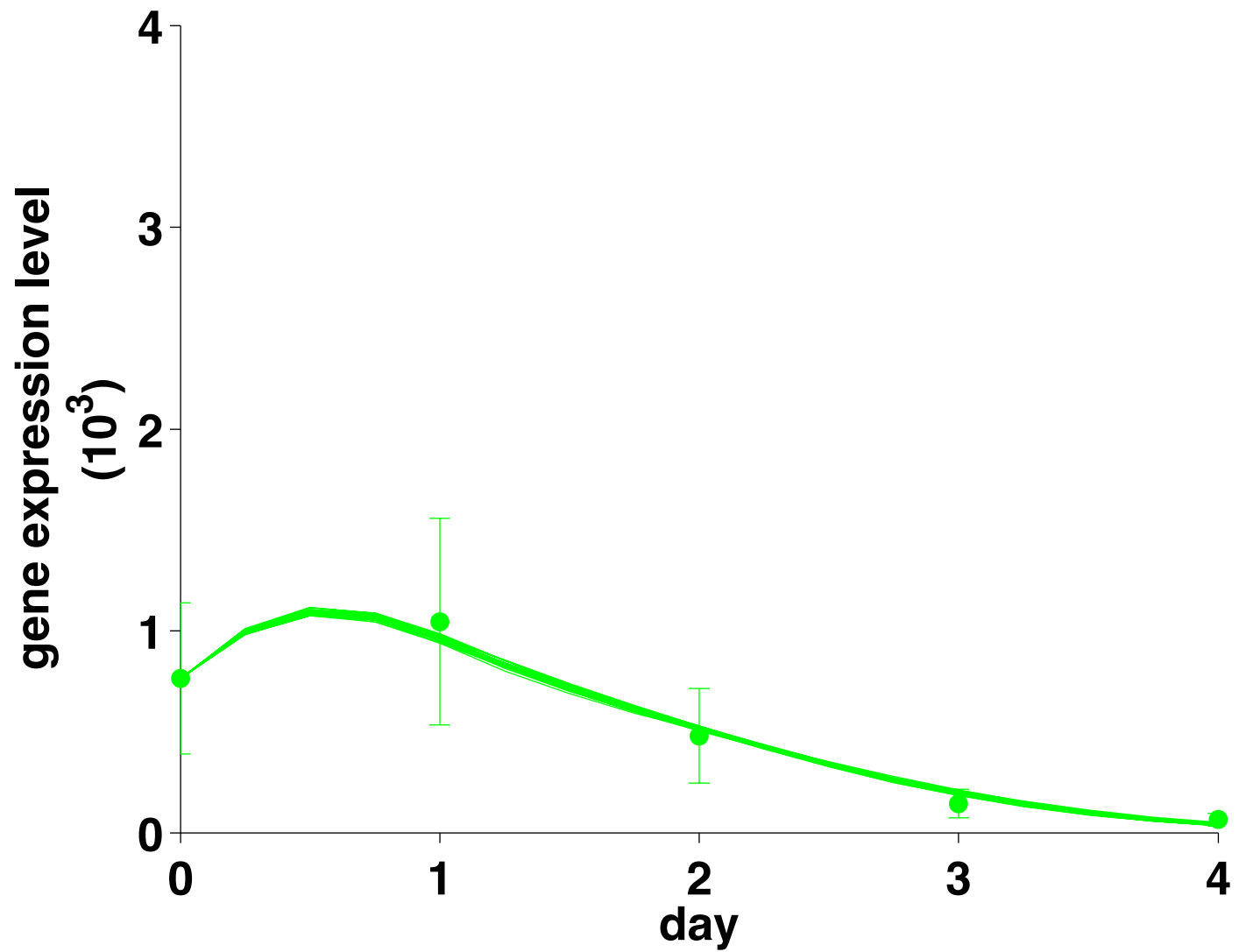

Supplement: S8 Fig — Dots: data points (adapted from Mingueneau et al. [17] according to our conversion stage to time); continuous lines: model predictions; bars: 95% intervals of confidence. (PDF) [file pone.0161260.s008.pdf]

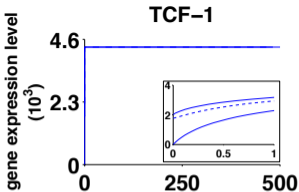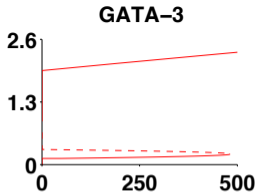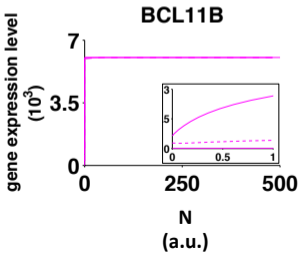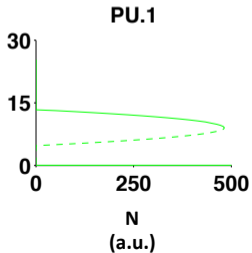

Supplement: S9 Fig — Blue lines: TCF-1; red lines: GATA-3; magenta lines: BCL11B; green lines: PU.1. Continuous lines: stable states; dashed lines: unstable states. (PDF) [file pone.0161260.s009.pdf]
