# Supplementary material for: Irreversibility of T-Cell Specification: Insights from Computational Modelling of a Minimal Network Architecture
Source: PLoS One. 2016 Aug 23;11(8):e0161260. doi: 10.1371/journal.pone.0161260 (PMC4995000; doi:10.1371/journal.pone.0161260)
Supplement: S5 Fig — (PDF) [file pone.0161260.s005.pdf]

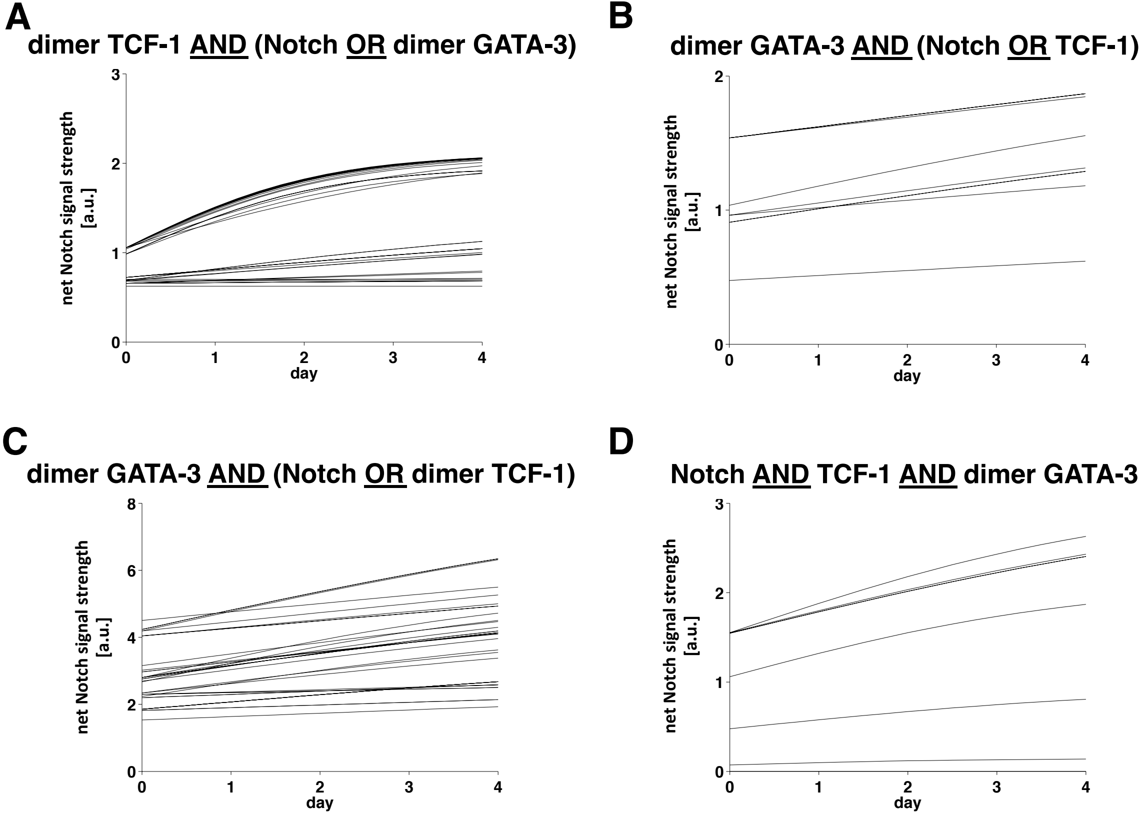
A
dimer TCF-1 AND (Notch OR dimer GATA-3)
net Notch signal strength [a.u.]
day
B
dimer GATA-3 AND (Notch OR TCF-1)
net Notch signal strength [a.u.]
day
C
dimer GATA-3 AND (Notch OR dimer TCF-1)
net Notch signal strength [a.u.]
day
D
Notch AND TCF-1 AND dimer GATA-3
net Notch signal strength [a.u.]
day
