# Supplementary material for: Irreversibility of T-Cell Specification: Insights from Computational Modelling of a Minimal Network Architecture
Source: PLoS One. 2016 Aug 23;11(8):e0161260. doi: 10.1371/journal.pone.0161260 (PMC4995000; doi:10.1371/journal.pone.0161260)
Supplement: S1 File — (PDF) [file pone.0161260.s010.pdf]

Supporting Text S1

Irreversibility of T-cell specification: insights from computational modelling of a minimal network architecture

Erica Manesso, Hao Yuan Kueh, George Freedman, Ellen V. Rothenberg, Carsten Peterson

Detailed Materials and Methods

Background

While networks accounting for the subdivision of embryonic tissue fates can be framed in Boolean logic terms[1], GRN accounting for signalling response kinetics are normally modelled with continuous functions. In applying these models to single-cell fate determination, a crucial difference is whether spatial partitioning or temporal features of a response are to be explained. When time-series are available, continuous models for GRN are a good compromise between (a) the necessity to quickly explore different architectures and to perform inference, well-covered by the logical models, and (b) the need to capture stochastic behaviour, accomplished only by single-molecule level models[2]. Inspired by topological models proposed in developmental studies[3,4,5,6,7,8], we exploited 32 combinatorial combinations to describe the dynamics of BCL11B as function of Notch signalling, TCF-1, and GATA-3. The results confirmed that a simple sum of these three components (i.e. Notch OR TCF-1 OR GATA-3) in BCL11B production rate is not enough to justify the delay in BCL11B versus the increase of TCF-1 and GATA-3, typical of a feed-forward motif[9], thus suggesting that complex interactions are needed. On the other hand, a simple AND interaction among Notch signalling, TCF-1, and GATA-3 failed to keep high levels BCL11B expressed when Notch signalling disappears.

The initial conditions on which our model was built come from published experimental data. The potential for GATA-3, TCF-1, BCL11B and PU.1 to act on each other is based on gene expression effects measured in acute perturbation experiments[10,11,12,13,14,15,16,17,18,19,20,21,22,23,24,25,26,27], while genome-wide binding profiles of GATA-3, PU.1, TCF-1 and more limited data for Notch binding indicate candidate sites where direct regulatory interactions may be mediated[20,27,28,29]. The proposed minimal model does not include all genes that are known to change in expression during the ETP-DN3a stages[29,30,31]. For example, both PU.1 and BCL11B receive additional regulatory inputs from Runx/CBFβ complexes[14,27,30,32,33]. However, RUNX transcription factors (especially RUNX1) are only slightly affected by Notch signals and change expression very little from ETP to DN3 stage 6 and have therefore been omitted. Also, GATA-3 and PU.1 are thought to interact at the protein level in a mutually antagonistic way[34,35,36,37,38]: however, the mechanisms regulating thresholds for this effect are not yet well understood, and it therefore has not been included. Also, the inclusion of additional genes that could act as intermediate links for certain regulatory relationships[30] could add biological detail to the framework of the model. However, this minimal model is robust with ranges of parameter values for which three key regulatory genes can mediate the irreversible change in state from multi-potent progenitors to committed T-cell precursors.

Model equations

The concentration levels of TCF-1, GATA-3, BCL11B, and PU.1 are denoted as $[T]$, $[G]$, $[B]$, and $[P]$ respectively. The Notch signalling is referred as $Notch$.

The concentration level of TCF-1 is described by the following dynamical equation:

$$\frac{1}{f_T} \cdot \frac{d[T]}{dt} = \frac{\eta_1 \cdot Notch + \eta_2 \cdot [T]^{n_{T,T}} + \eta_3 \cdot [G]^{n_{T,G}}}{1 + \eta_1 \cdot Notch + \eta_2 \cdot [T]^{n_{T,T}} + \eta_3 \cdot [G]^{n_{T,G}} + \eta_4 \cdot [P]^{n_{T,P}}} - \gamma_T \cdot [T] \tag{S1}$$

where: the η_i (i=1, ..., 4) modulate the production of TCF-1; γ_T is the degradation rate (day^{-1}) scaled by the factor f_T; $n_{T,T}, n_{T,G}$, and $n_{T,P}$ are the Hill coefficients (set to 1). We thus define the vector of parameters describing TCF-1 dynamics as Θ_T=[$\eta_1, \eta_2, \eta_3, \eta_4, \gamma_T, n_{T,T}, n_{T,G}, n_{T,P}, f_T$].

Similarly, the concentration level of GATA-3 is described by:

$$\frac{1}{f_G} \cdot \frac{d[G]}{dt} = \frac{\delta_1 \cdot Notch + \delta_2 \cdot [T]^{n_{G,T}}}{1 + \delta_1 \cdot Notch + \delta_2 \cdot [T]^{n_{G,T}} + \delta_3 \cdot [P]^{n_{G,P}}} - \gamma_G \cdot [G] \tag{S2}$$

where: the δ_i (i=1, ..., 3) shape the production of GATA-3; γ_G is the degradation rate (day^{-1}) scaled by the factor f_G; $n_{G,T}$ and $n_{G,P}$ are the Hill coefficients (set to 1). The vector of parameters for GATA-3 dynamics is then Θ_G=[$\delta_1, \delta_2, \delta_3, \gamma_G, n_{G,T}, n_{G,P}, f_G$].

Depending on how the Notch signalling, TCF-1, and GATA-3 collaborate to activate BCL11B, the concentration level of BCL11B is described by one of the following equations which cover all the possible combinatorial interactions among the three players.

1. Notch OR TCF-1 OR GATA-3 activate BCL11B

$$\frac{1}{f_{B,1}} \cdot \frac{d[B]}{dt} = \frac{\kappa_{1,1} \cdot Notch + \kappa_{2,1} \cdot [T]^{n_{B,T,1}} + \kappa_{3,1} \cdot [G]^{n_{B,G,1}}}{1 + \kappa_{1,1} \cdot Notch + \kappa_{2,1} \cdot [T]^{n_{B,T,1}} + \kappa_{3,1} \cdot [G]^{n_{B,G,1}}} - \gamma_{B,1} \cdot [B] \tag{S3}$$

2. (Notch AND TCF-1) OR GATA-3 turn on BCL11B

$$\frac{1}{f_{B,2}} \cdot \frac{d[B]}{dt} = \frac{\kappa_{1,2} \cdot Notch \cdot [T]^{n_{B,T,2}} + \kappa_{2,2} \cdot [G]^{n_{B,G,2}}}{1 + \kappa_{1,2} \cdot Notch \cdot [T]^{n_{B,T,2}} + \kappa_{2,2} \cdot [G]^{n_{B,G,2}}} - \gamma_{B,2} \cdot [B] \tag{S4}$$

3. (Notch AND GATA-3) OR TCF-1 switch on BCL11B

$$\frac{1}{f_{B,3}} \cdot \frac{d[B]}{dt} = \frac{\kappa_{1,3} \cdot Notch \cdot [G]^{n_{B,G,3}} + \kappa_{2,3} \cdot [T]^{n_{B,T,3}}}{1 + \kappa_{1,3} \cdot Notch \cdot [G]^{n_{B,G,3}} + \kappa_{2,3} \cdot [T]^{n_{B,T,3}}} - \gamma_{B,3} \cdot [B] \tag{S5}$$

4. Notch OR (TCF-1 AND GATA-3) trigger BCL11B

$$\frac{1}{f_{B,4}} \cdot \frac{d[B]}{dt} = \frac{\kappa_{1,4} \cdot Notch + \kappa_{2,4} \cdot [T]^{n_{B,T,4}} \cdot [G]^{n_{B,G,4}}}{1 + \kappa_{1,4} \cdot Notch + \kappa_{2,4} \cdot [T]^{n_{B,T,4}} \cdot [G]^{n_{B,G,4}}} - \gamma_{B,4} \cdot [B] \tag{S6}$$

5. Notch AND (TCF-1 OR GATA-3) activate BCL11B

$$\frac{1}{f_{B,5}} \cdot \frac{d[B]}{dt} = \frac{Notch \cdot (\kappa_{1,5} \cdot [T]^{n_{B,T,5}} + \kappa_{2,5} \cdot [G]^{n_{B,G,5}})}{1 + Notch \cdot (\kappa_{1,5} \cdot [T]^{n_{B,T,5}} + \kappa_{2,5} \cdot [G]^{n_{B,G,5}})} - \gamma_{B,5} \cdot [B] \tag{S7}$$

6. TCF-1 AND (Notch OR GATA-3) turn on BCL11B

$$\frac{1}{f_{B,6}} \cdot \frac{d[B]}{dt} = \frac{[T]^{n_{B,T,6}} \cdot (\kappa_{1,6} \cdot Notch + \kappa_{2,6} \cdot [G]^{n_{B,G,6}})}{1 + [T]^{n_{B,T,6}} \cdot (\kappa_{1,6} \cdot Notch + \kappa_{2,6} \cdot [G]^{n_{B,G,6}})} - \gamma_{B,6} \cdot [B] \quad \text{(S8)}$$

7. GATA-3 AND (Notch OR TCF-1) switch on BCL11B

$$\frac{1}{f_{B,7}} \cdot \frac{d[B]}{dt} = \frac{[G]^{n_{B,G,7}} \cdot (\kappa_{1,7} \cdot Notch + \kappa_{2,7} \cdot [T]^{n_{B,T,7}})}{1 + [G]^{n_{B,G,7}} \cdot (\kappa_{1,7} \cdot Notch + \kappa_{2,7} \cdot [T]^{n_{B,T,7}})} - \gamma_{B,7} \cdot [B] \quad \text{(S9)}$$

8. Notch AND TCF-1 AND GATA-3 trigger BCL11B

$$\frac{1}{f_{B,8}} \cdot \frac{d[B]}{dt} = \frac{\kappa_{1,8} \cdot Notch \cdot [T]^{n_{B,T,8}} \cdot [G]^{n_{B,G,8}}}{1 + \kappa_{1,8} \cdot Notch \cdot [T]^{n_{B,T,8}} \cdot [G]^{n_{B,G,8}}} - \gamma_{B,8} \cdot [B] \quad \text{(S10)}$$

The $\kappa_{i,j}$ (i=1, ..., 3; j=1, ..., 8) modulate the production of BCL11B; γ_B is the degradation rate (day^{-1}) scaled by the factor f_B; $n_{B,T,j}$ and $n_{B,G,j}$ are the Hill coefficients, assuming the value of 1 in case of a monomer, 2 in case of a dimer. We thus define the vector of parameters depicting BCL11B dynamics as Θ_B=[$\kappa_{i,j}, \gamma_B, n_{B,T,j}, n_{B,G,j}, f_B$]. As the Hill coefficients $n_{B,T,j}$ and $n_{B,G,j}$ can assume 2 possible values (either 1 or 2), the total number of configurations for the BCL11B production rate are 32 (8×2^2).

The concentration level of PU.1 is described by:

$$\frac{1}{f_P} \cdot \frac{d[P]}{dt} = \frac{\alpha_1 \cdot [P]^{n_{P,P}}}{1 + \alpha_1 \cdot [P]^{n_{P,P}} + \alpha_2 \cdot [T]^{n_{P,T}} + \alpha_3 \cdot [G]^{n_{P,G}} + \alpha_4 \cdot [B]^{n_{P,B}}} - \gamma_P \cdot [P] \quad \text{(S11)}$$

where: the α_i (i=1, ..., 4) shape the production of PU.1; γ_P is the degradation rate (day^{-1}); $n_{P,P}$, $n_{P,T}$, $n_{P,G}$. and $n_{P,B}$ are the Hill coefficients (set to 1); f_P is the uniforming parameter. The vector of parameters for PU.1 dynamics is then Θ_P=[$\alpha_1, \alpha_2, \alpha_3, \alpha_4, \gamma_P, n_{P,P}, n_{P,T}, n_{P,G}, n_{P,B}, f_P$].

Parameter inference

To determine the parameter space for TCF-1, GATA-3, BCL11B, and PU.1, we first calculated the derivate of each gene expression profile. For this purpose, two independent methods (providing similar results) were applied: (1) a parametric approach; (2) an algorithm based on a stochastic regularization method[39]. The first method consisted in fitting each gene profile with a parametric function and determining the derivative from the best parametric function. The second approach is based on the concept of deconvolution, which allows us to simultaneously perform both data regularization and calculation of the derivative.

We then decided to split the parameter estimation problem in two parts: the first considered PU.1 as known from smoothing procedure (i.e. PU.1 as forcing function) and focused on the estimation of the parameters characterising TCF-1, GATA-3, and BCL11B dynamics; the latter fixed TCF-1, GATA-3, and BCL11B to their respective smoothing profiles (that is, TCF-1, GATA-3, and BCL11B as forcing functions) and aimed to determine PU.1 parameter values.

TCF-1, GATA-3, and BCL11B parameters. Once the derivative was calculated, each equation describing the dynamics of TCF-1, GATA-3, and BCL11B (Eqs. S1-S10 was re-written to extrapolate the profile of Notch signalling (generally known except for its multiplying parameter). E.g. for TCF-1

$$\eta_1 \cdot Notch = -\eta_2 \cdot [T]^{n_{T,T}} - \eta_3 \cdot [G]^{n_{T,G}} - \frac{(1 + \eta_4 \cdot [P]^{n_{T,P}} \cdot (\frac{d[T]}{dt} \cdot \frac{1}{f_T} + \gamma_T \cdot [T])}{\frac{d[T]}{dt} \cdot \frac{1}{f_T} + \gamma_T \cdot [T] - 1} \tag{S12}$$

The requirement that Notch signalling activity is positive in ETP-DN3a stages[40,41] implied a number of inequalities that led to define the lower and upper bounds for most of the unknown parameters. For TCF-1 parameters Θ_T the inequalities are

$$0 < \eta_2 < \frac{-\eta_3 \cdot [G]^{n_{T,G}} - a \cdot (1 + \eta_4 \cdot [P]^{n_{T,P}}}{[T]^{n_{T,T}}} \tag{S13}$$

$$0 < \eta_3 < \frac{-a \cdot (1 + \eta_4 \cdot [P]^{n_{T,P}}}{[G]^{n_{T,G}}} \tag{S14}$$

$$0 < \gamma_T < \frac{1}{[T]} \tag{S15}$$

$$f_T > \frac{\frac{d[T]}{dt}}{1 - \gamma_T \cdot [T]} \tag{S16}$$

where a is

$$a = \frac{\frac{d[T]}{dt} \cdot \frac{1}{f_T} + \gamma_T \cdot [T]}{\frac{d[T]}{dt} \cdot \frac{1}{f_T} + \gamma_T \cdot [T] - 1} \tag{S17}$$

As for some parameters the bounds were not defined by the inequalities (e.g. upper bound for f_T), we performed a preliminary exploration of the parameter space by the Monte Carlo method to determine the missing bounds. This step was performed separately for the sets Θ_T, Θ_G, and Θ_B. The measurements of GATA-3 half-life in the range 2-4 hours (Figure S1 - panel B) allowed us to refine the bounds for the degradation rate parameter γ_G. To determine Θ_B each combinatorial configuration for BCL11B production was analysed. The steps described above were run in parallel for the two regularisation methods and the bounds were then merged so that the largest parameter space was defined.

We then modelled the Notch signalling as a sigmoidal function

$$Notch = \frac{N}{1 + e^{-\alpha \cdot t}} \tag{S18}$$

where N is the maximum level of Notch signalling and α is the increasing rate (day^{-1}). We denote as Θ_N the parameter vector [N,α].

Once defined the bounds for the parameter space, a constrained multi-objective optimisation approach based on the Pareto frontier[42] was applied to determine the best parameter configurations

for Θ_T, Θ_G, Θ_B, and Θ_N that fit simultaneously TCF-1, GATA-3, and BCL11B time series (for each of the 32 configurations of BCL11B dynamics). The three different objectives were referred to the sum of the squared residuals of TCF-1, GATA-3, and BCL11B respectively. As in general Notch signalling is generally known except for its multiplying parameters (i.e. η_1, δ_1, $\kappa_{1,j}$, j=1, . . . , 8), we fixed N to 1 during the optimisation. The constraint included the Notch signalling increase between ETP and DN3a stages by a factor in the range [1,3.5]. To allow the parameter estimation, the number of data to fit was increased by interpolation in a uniform grid. A multi-objective optimization approach was preferred to a single global optimization to have better fit of the single gene profiles.

The resulting configurations were then filtered according to the 95% intervals of confidence (assuming an error in the data of 25%). Table S2 sums up the description of the parameters mentioned above.

PU.1 parameters. The equation describing the dynamics of PU.1 (Eq. S11 was re-written to extrapolate the profile of BCL11B (known expect for its multiplying parameter)

$$\alpha_4 \cdot [B]^{n_{P,B}} = -1 - \alpha_2 \cdot [T]^{n_{P,T}} - \alpha_3 \cdot [G]^{n_{P,G}} - \frac{\alpha_1 \cdot [P]^{n_{P,P}} \cdot (\frac{d[P]}{dt} \cdot \frac{1}{f_P} + \gamma_P \cdot [P] - 1)}{\frac{d[P]}{dt} \cdot \frac{1}{f_P} + \gamma_P \cdot [P]} \quad \text{(S19)}$$

The requirement that BCL11B be positive implied a number of inequalities that led to define most the lower and upper bounds for the unknown parameters in Θ_P. Monte Carlo method was applied to determine the missing bounds. n (order 10^2) runs of simulated annealing under the constraints $\alpha_4 < \alpha_2$ and $\alpha_4 < \alpha_3$ (i.e. decreased inhibition power of PU.1 by BCL11B versus TCF-1 and GATA-3) were performed. Finally the 95% intervals of confidence filtering provided the optimal parameter values for the vector Θ_P.

Data regularisations, Monte Carlo simulations, multi-objective optimisation, simulated annealing, and analysis were performed by MATLAB 2014a software (The Mathworks, Natick, MA).

Steady-state analysis

To ensure reaching high levels of TCF-1, GATA-3, and BCL11B, but low levels of PU.1, the entire network was simulated to reach the steady state by taking separately each of the n parameter sets for PU.1 dynamics (Θ_P) resulting from the simulated annealing. The parameters describing TCF-1, GATA-3, BCL11B, and Notch signals, i.e. Θ_T, Θ_G, Θ_B, and Θ_N, were fixed to their best values (best in terms of adherence to the data, i.e. parameter values that minimise the mean squared differences between model prediction and data points).

Bifurcation analysis

Bifurcation analysis with respect to the maximum value of Notch signalling (N, arbitrary unit) was performed by the software XPP-Aut, which contains an interface to AUTO-07p (Concordia University, Montreal, Canada). The kinetic parameters were set to the best configuration values (best in terms of adherence to the data).

References

1. Peter IF, Faure E, Davidson EH. Predictive computation of genomic logic processing functions in embryonic development *Proc Natl Acad Sci U S A* 2012; **109**: 16434-16442.

2. Karlebach G, Shamir R. Modelling and analysis of gene regulatory networks. *Nat Rev Mol Cell Biol* 2008; **9**: 770-780.

3. Davidson EH. Emerging properties of animal gene regulatory networks. *Nature* 2010; **468**: 911-920.

4. Cotterell J, Sharpe J. An atlas of gene regulatory networks reveals multiple three-gene mechanisms for interpreting morphogen gradients. *Mol Syst Biol* 2010; **6**: 425.

5. François P, Siggia ED. Predicting embryonic patterning using mutual entropy fitness and in silico evolution. *Development* 2010; **137**: 2385-2395.

6. Cotterell J, Sharpe J. Mechanistic explanations for restricted evolutionary paths that emerge from gene regulatory networks. *PLoS One* 2013; **8**: e61178.

7. May G, Soneji S, Tipping AJ, Teles J, McGowan SJ, Wu M *et al.* Dynamic analysis of gene expression and genome-wide transcription factor binding during lineage specification of multipotent progenitors. *Cell Stem Cell* 2013; **13**: 754-768.

8. Munteanu A, Cotterell J, Solé RV, Sharpe J. Design principles of stripe-forming motifs: the role of positive feedback. *Sci Rep* 2014; **4**: 5003.

9. Mangan S, Zaslaver A, Alon U. The coherent feedforward loop serves as a sign-sensitive delay element in transcription networks. *J Mol Biol* 2003; **334**: 197-204.

10. Del Real-Morales M, Rothenberg EV. Architecture of a lymphomyeloid developmental switch controlled by Pu.1, Notch and Gata3. *Development* 2013; **140**: 1207-1219.

11. Okuno Y, Huang G, Rosenbauer F, Evans EK, Radomska HS, Iwasaki H *et al.* Potential autoregulation of transcription factor Pu.1 by an upstream regulatory element. *Mol Cell Biol* 2005; **25**: 2832-2845.

12. Taghon TN, David ESS, Zúñiga-Pflücker JC, Rothenberg EV. Delayed, asynchronous, and reversible T-lineage specification induced by Notch/Delta signalling. *Genes Dev* 2005; **19**: 965-978.

13. Franco CB, Scripture-Adams DD, Proekt I, Taghon T, Weiss AH, Yui MA *et al.* Notch/Delta signalling constrains reengineering of pro-T cells by Pu.1. *Proc Natl Acad Sci U S A* 2006; **103**: 11993-11998.

14. Guo Y, Maillard I, Chakraborti S, Rothenberg EV, Speck NA. Core binding factors are necessary for natural killer cell development and cooperate with Notch signalling during T-cell specification. *Blood* 2008; **112**: 480-492.

15. Weerkamp F, Luis TC, Naber BAE, Koster EEL, Jeannotte L, van Dongen JJM *et al.* Identification of Notch target genes in uncommitted T-cell progenitors: no direct induction of a T-cell specific gene program. *Leukemia* 2006; **20**: 1967-1977.

16. Taghon T, Yui MA, Rothenberg EV. Mast cell lineage diversion of T lineage precursors by the essential T cell transcription factor GATA-3. *Nat Immunol* 2007; **8**: 845-855.

17. Tydell CC, David-Fung ESD, Moore JE, Rowen L, Taghon T, Rothenberg EV. Molecular dissection of prethymic progenitor entry into the T lymphocyte developmental pathway. *J Immunol* 2007; **179**: 421-438.

18. Van de Walle I, De Smet G, De Smedt M, Vandekerckhove B, Leclercq G, Plum, J *et al.* An early decrease in Notch activation is required for human TCR-$\alpha\beta$ lineage differentiation at the expense of TCR-$\gamma\delta$ T cells. *Blood* 2009; **113**: 2988-2998.

19. Ikawa T, Hirose S, Masuda K, Kakugawa K, Satoh R, Shibano-Satoh A *et al.* An essential developmental checkpoint for production of the T cell lineage. *Science* 2010; **329**: 93-96.

20. Li P, Burke S, Wang J, Chen X, Ortiz M, Lee SCC *et al.* Reprogramming of T cells to natural killer-like cells upon *Bcl11b* deletion. *Science* 2010; **329**: 85-89.

21. Yui MA, Feng N, Rothenberg EV. Fine-scale staging of T-cell lineage commitment in adult mouse thymus. *J Immunol* 2010; **185**: 284-293.

22. Leddin M, Perrod C, Hoogenkamp M, Ghani S, Assi S, Heinz S *et al.* Two distinct autoregulatory loops operate at the Pu.1 locus in B cells and myeloid cells. *Blood* 2011; **117**: 2827-2838.

23. Weber BN, Chi AWS, Chavez A, Yashiro-Ohtani Y, Yang Q, Shestova O *et al.* A critical role for Tcf-1 in T-lineage specification and differentiation. *Nature* 2011; **476**: 63-68.

24. Kueh HY, Rothenberg EV. Regulatory gene network circuits underlying T-cell development from multi-potent progenitors. *Wiley Interdiscip Rev Syst Biol Med* 2012; **4**: 79-102.

25. García-Ojeda ME, Klein Wolterink RGJ, Lemaître F, Richard-Le Goff O, Hasan M, Hendriks RW *et al.* Gata-3 promotes T-cell specification by repressing B-cell potential in pro-T cells in mice. *Blood* 2013; **121**: 1749-1759.

26. Zarnegar MA, Rothenberg EV. Ikaros represses and activates Pu.1 cell-type-specifically through the multifunctional Sfpi1 URE and a myeloid specific enhancer. *Oncogene* 2012; **31**: 4647-4654.

27. Li L, Zhang JA, Dose M, Kueh HY, Mosadeghi R, Gounari F *et al.* A far downstream enhancer for murine Bcl11b controls its T-cell specific expression. *Blood* 2013; **122**: 902-911.

28. Geimer Le Lay AS, Oravecz A, Mastio J, Jung C, Marchal P, Ebel C, *et al.* The tumor suppressor Ikaros shapes the repertoire of Notch target genes in T cells. *Science signaling* 2014; **7**: ra28.

29. Zhang JA, Mortazavi A, Williams BA, Wold BJ, Rothenberg EV. Dynamic transformations of genome-wide epigenetic marking and transcriptional control establish T-cell identity. *Cell* 2012; **149**: 467-482.

30. Yui MA, Rothenberg EV. Developmental gene networks: a triathlon on the course to T cell identity. *Nature Reviews Immunology* 2014; **14**: 529-545.

31. Mingueneau M, Kreslavsky T, Gray D, Heng T, Cruse R, Ericson J *et al.* The transcriptional landscape of $\alpha\ \beta$ T cell differentiation. *Nat Immunol* 2013; **14**: 619-632.

32. Huang G, Zhang P, Hirai H, Elf S, Yan X, Chen Z *et al.* Pu.1 is a major downstream target of Aml1 (Runx1) in adult mouse hematopoiesis. *Nat Genet* 2008; **40**: 51-60.

33. Zarnegar MA, Chen J, Rothenberg EV. Cell-type-specific activation and repression of Pu.1 by a complex of discrete, functionally specialized cis-regulatory elements. *Mol Cell Biol* 2010; **30**: 4922-4939.

34. Zhang P, Behre G, Pan J, Iwama A, Wara-Aswapati N, Radomska HS et al. Negative cross-talk between hematopoietic regulators: Gata proteins repress Pu.1. *Proc Natl Acad Sci U S A* 1999; **96**: 8705-8710.

35. Cantor AB, Orkin SH. Transcriptional regulation of erythropoiesis: an affair involving multiple partners. *Oncogene* 2002; **21**: 3368-3376.

36. Stopka T, Amanatullah DF, Papetti M, Skoultchi AI. Pu.1 inhibits the erythroid program by binding to Gata-1 on DNA and creating a repressive chromatin structure. *The EMBO journal* 2005; **24**: 3712-3723.

37. Liew CW, Rand KD, Simpson RJY, Yung WW, Mansfield RE, Crossley M, *et al.* Molecular analysis of the interaction between the hematopoietic master transcription factors Gata-1 and Pu.1. *J Biol Chem* 2006; **281**: 28296-28306.

38. 59. Chang HCC, Han L, Jabeen R, Carotta S, Nutt SL, Kaplan MH. Pu.1 regulates TCR expression by modulating Gata-3 activity. *J Immunol* 2009; **183**: 4887-4894.

39. De Nicolao G, Sparacino G, Cobelli C. Nonparametric input estimation in physiological systems: problems, methods, and case studies. *Automatica* 1997; **33**: 851-870.

40. Kleinmann E, Geimer Le Lay AS, Sellars M, Kastner P, Chan S. Ikaros represses the transcriptional response to Notch signalling in T-cell development. *Mol Cell Biol* 2008; **28**: 7465-7475.

41. Oh P, Lobry C, Gao J, Tikhonova A, Loizou E, Manent J *et al.* In vivo mapping of notch pathway activity in normal and stress hematopoiesis. *Cell Stem Cell* 2013; **13**: 190-204.

42. Kalyanmoy D, Pratap A, Agarwal S, Meyarivan T. A fast and elitist multi-objective genetic algorithm: NSGA-II. *IEEE Transaction on Evolutionary Computation* 2002; **6**: 181-197.

43. Scripture-Adams DD, Damle SS, Li L, Elihu KJ, Qin S, Arias AM *et al.* Gata-3 dose-dependent checkpoints in early T cell commitment. *J Immunol* 2014; **193**: 3470-3491.

Tables

Table S1 Means and Standard Deviations (SD) for the winning parameters of PU.1 dynamics.

Parameters	Description	Mean	SD
α_1	affinity for PU.1 binding on *Pu.1* locus	1.5E-03	2.2E-03
α_2	affinity for TCF-1 binding on *Pu.1* locus	1.3E-03	1.2E-03
α_3	affinity for GATA-3 binding on *Pu.1* locus	5.3E-03	6.6E-03
α_4	affinity for BCL11B binding on *Pu.1* locus	1.2E-03	1.2E-03
γ_P	PU.1 degradation rate	1.3E-04	7.3E-05
f_P	PU.1 scaling factor	5.4E+04	6.0E+04
half-life PU.1	(hour)	4.0E+00	1.9E-01

Table S2 Parameter description for TCF-1, GATA-3 and BCL11B dynamics and Notch signalling profile.

Parameters	Description
η_1	Notch effect on TCF-1
η_2	affinity for TCF-1 binding on *Tcf7* locus
η_3	affinity for GATA-3 binding on *Tcf7* locus
η_4	affinity for PU.1 binding on *Tcf7* locus
γ_T	TCF-1 degradation rate
f_T	TCF-1 scaling factor
δ_1	Notch effect on GATA-3
δ_2	affinity for TCF-1 binding on *Gata3* locus
δ_3	affinity for PU.1 binding on *Gata3* locus
γ_G	GATA-3 degradation rate
f_G	GATA-3 scaling factor
$\kappa_{i,j}$	parameters that regulate the production of BCL11B
γ_B	BCL11B degradation rate
f_B	BCL11B scaling factor
N	Maximum level of Notch signalling
α	Increasing rate of Notch signalling
